# Free mRNA in excess upon polysome dissociation is a scaffold for protein multimerization to form stress granules

**DOI:** 10.1093/nar/gku582

**Published:** 2014-07-10

**Authors:** Ouissame Bounedjah, Bénédicte Desforges, Ting-Di Wu, Catherine Pioche-Durieu, Sergio Marco, Loic Hamon, Patrick A. Curmi, Jean-Luc Guerquin-Kern, Olivier Piétrement, David Pastré

**Affiliations:** 1 Institut National de la Santé et de la Recherche Médicale (INSERM), UMR829; Université Evry-Val d'Essonne, Evry 91025, France; 2 Institut Curie, INSERM, U759, 91405 Orsay cedex, France; 3 Centre National de la Recherche Scientifique (CNRS), UMR 8126; University of Paris Sud, 94805 Villejuif, France

## Abstract

The sequence of events leading to stress granule assembly in stressed cells remains elusive. We show here, using isotope labeling and ion microprobe, that proportionally more RNA than proteins are present in stress granules than in surrounding cytoplasm. We further demonstrate that the delivery of single strand polynucleotides, mRNA and ssDNA, to the cytoplasm can trigger stress granule assembly. On the other hand, increasing the cytoplasmic level of mRNA-binding proteins like YB-1 can directly prevent the aggregation of mRNA by forming isolated mRNPs, as evidenced by atomic force microscopy. Interestingly, we also discovered that enucleated cells do form stress granules, demonstrating that the translocation to the cytoplasm of nuclear prion-like RNA-binding proteins like TIA-1 is dispensable for stress granule assembly. The results lead to an alternative view on stress granule formation based on the following sequence of events: after the massive dissociation of polysomes during stress, mRNA-stabilizing proteins like YB-1 are outnumbered by the burst of nonpolysomal mRNA. mRNA freed of ribosomes thus becomes accessible to mRNA-binding aggregation-prone proteins or misfolded proteins, which induces stress granule formation. Within the frame of this model, the shuttling of nuclear mRNA-stabilizing proteins to the cytoplasm could dissociate stress granules or prevent their assembly.

## INTRODUCTION

In eukaryotic cells, the mechanism leading to the formation of mRNA-containing granules remains a matter of debate (1,2). These granules comprise germ granules in germ cells (3), RNA granules in neurons (4), both cargoes for mRNA transport or segregation; P-bodies, possible centers of mRNA degradation (5); and stress granules, micrometric mRNA aggregates formed under stress conditions (6,7). A remarkable aspect of RNA-containing granules is the absence of encapsulating membranes which leaves RNA and associated RNA-binding proteins free to shuttle in and out of granules in a dynamical equilibrium rendering such aggregates, by nature, unstable (8). For this reason, the isolation of RNA-containing granules from cells and their further characterization *in vitro* remain an issue.

Recent advances however have been achieved to probe the composition of RNA granule-like structures formed *in vitro* (9). The authors discovered that an isoxazole derivative makes a microcrystalline precipitate which co-precipitates RNA-binding proteins enriched in repetitive low-complexity (LC) domains with significant overlap to the constituents of RNA granules. The precipitated proteins appeared in turn to provide a platform which can recruit and retain selectively RNA with long 3′UTR (9,10). From these results, it was proposed that the aggregation of LC domain-enriched proteins is a critical event in RNA-granule formation. This hypothesis is sound in the case of stress granules since mRNA-binding proteins displaying self-attracting LC sequences (11) or ‘prion-like’ domains such as TIA-1 (12), CIRP (13), G3BP (14), CPEB1 (15) and FUS (16) were found enriched in these granules. In addition, overexpressing these proteins (TIA-1 (12), CIRP (13), G3BP (14) and CPEB1 (15)) triggers the formation of RNA granules without any additional stimulus. As many RNA-binding proteins actively shuttle between the nucleus and the cytoplasm (17) like TIA-1 (18), their translocation from the nucleus to the cytoplasm could be one of the potential mechanism by which stress granules may appear after stress (13,19).

Albeit self-attracting RNA-binding proteins play a key role in the aggregation process, many reports have also shown that the suppression of translation (7,20) and especially the dissociation of polysomes are mandatory for stress granules assembly (21). In most cases, polysome dissociation occurs rapidly during stress via the phosphorylation of eIF2α, even if other routes exist (22). An excess of free mRNA could then be the early event for secondary recruitment of specific stress granule proteins. In line with this hypothesis, cycloheximide, which keeps ribosomes loaded onto mRNA by blocking translation elongation, dissociates stress granules, while puromycin, which unlocks ribosomes from mRNA, favors stress granule assembly (21,23). The requirement of nonpolysomal mRNA to form stress granules makes sense since polysomal mRNAs is protected from aggregation by ribosomes which are known for their intrinsic stability and self-repulsion (24).

To decipher the mechanism of stress granule assembly, we performed a series of novel experiments, which provide critical information. (i) We used dynamic secondary ion mass spectrometry ((nanoSIMS), (25)) in normal rat kidney (NRK) cells exposed to arsenite to reveal at a submicrometric resolution the putative RNA enrichment compared to proteins in stress granules. (ii) Via the delivery of exogenous nucleic acids to cells, we probed whether or not stress granule assembly could result from a nucleic acid/protein imbalance in the cytoplasm. (iii) We explored using atomic force microscopy (AFM) whether a cytoplasmic mRNA-binding protein like YB-1 (26) can directly prevent the formation of mRNA:TIA-1 granules via its binding to mRNA. (iv) We determined using enucleated cells whether the shuttling of prion-like mRNA-binding proteins like TIA-1 from the nucleus to the cytoplasm is required for stress granule assembly. (v) We finally explored an alternative model in which the relocation of nucleocytoplasmic shuttling proteins in the cytoplasm like HuR (27) could dissociate stress granules or prevent their assembly rather than induce it.

## MATERIALS AND METHODS

### Chemical and reagents

VER-155008, quercetin, PKR inhibitor (PKRi or c16), MG132, SP600125, H-89, U1026, PD0325901 were purchased from Sigma. SB203580 and CDKi (CAS 40254-90-8) were purchased from Invitrogen and Santa-Cruz, respectively. ^15^N-enriched uridine (2-^15^N) was obtained from Cambridge corporation laboratories.

### Electron microscopy

NRK cells grown on glass coverslips were fixed for 15 min at 37°C with 1.6% glutaraldehyde in 0.1 M phosphate buffer, pH 7.3. Cells were then dehydrated using increasing concentrations of ethanol and flat-embedded in Epon (Embed-812 Embedding kit #14120, EMS). Collodion-carbon-coated copper grids were used to collect ultrathin sections of 100-nm thickness. The sections were then stained with 2% uranyl acetate aqueous solution and analyzed using a Zeiss 902 transmission electron microscope. For the immuno-localization of YB-1, NRK cells grown on glass coverslips were fixed for 2 h at room temperature in 4% paraformaldehyde (PFA) and 0.25% glutaraldehyde in 0.1 M phosphate buffer, pH 7.3 and postfixed for 1 h in 4% PFA. At this point, the following steps were performed on ice. Cells were dehydrated as previously. Samples were flat-embedded in LR White embedding medium (#14380, EMS). Polymerization was performed at 50°C for 24 h. Immuno-labeling was performed upon ultrathin sections of LR White-embedded samples collected on collodion-carbon coated nickel grids. Sections were first washed in phosphate buffered saline (PBS), incubated in 50 mM NH_4_Cl for 20 min and in a blocking solution for 30 min. The hybridization step took place with blocking solution containing a rabbit anti-YB-1 (produced as described in Davydova *et al.* (28)) for 1 h (1:4000 dilution). After extensive washing with PBS, 1% bovine serum albumin (BSA), grids were incubated for 1 h with anti-rabbit secondary antibody coupled to 15-nm gold particles at a dilution of 1/25 (EM.GAR15, BB International, Cardiff, UK). After extensive washes in PBS, labeling was fixed with glutaraldehyde 2.5% in PBS. Samples were then washed in distilled water, counterstained with 2% uranyl acetate aqueous solution and observed in bright-field mode, using a Zeiss 902 transmission electron microscope. Images were acquired using a Megaview III CCD camera and the iTEM software (Olympus Soft Imaging Solution).

### Secondary ion mass spectroscopy (nanoSIMS)

NRK cells were grown on glass coverslips in the presence of 100 μM ^15^N-uridine for 16 h. Cells were washed rapidly with PBS at 37°C to remove unincorporated ^15^N-uracyl and then exposed to 300 μM arsenite for 45 min when specified. Samples were embedded in Epon and prepared as indicated for electron microscopy. Thin sections (150 nm) were deposited on clean silicon chips and then inserted into a NanoSIMS-50 Ion microprobe (CAMECA, Gennevilliers, France) operating in scanning mode (25). For the present study, by using a Cs^+^ primary ion beam tightly focused to a typical probe size of ≈85 nm (distance between 16 and 84% of peak intensity from a line scan), four secondary ion species (^12^C^−^, ^12^C^14^N^−^, ^12^C^15^N^−^ and ^31^P^−^) were monitored simultaneously. The primary beam steps over the surface of the sample to create images of the selected ion species. After careful Cs^+^ ion implantation to get steady state ion emission, the acquisition was carried out using multiframe mode. The primary beam intensity was 1 pA and the raster size was from 20 to 35 μm in order to image a whole cell with an image definition of 512 × 512 pixels. With a dwell time of 2 ms per pixel, up to 15 frames were acquired and the total analysis time was 2 h. Image processing was performed using the ImageJ software (29). First, multiframe images were properly aligned using ^12^C^14^N^−^ images as reference before a summed image was obtained for each ion species. ^15^N:^14^N ratios were established from ^12^C^14^N and ^12^C^15^N data. A sample containing no labeled cells was used as working reference for adjusting the detectors prior to quantification of ^15^N:^14^N ratios. Further, a check was performed on the resin area surrounding the observed cells. The final ^15^N:^14^N ratio is displayed in Hue-Saturation-Intensity (HSI) mode. These HSI color images were generated using OpenMIMS, an ImageJ plugin developed by Claude Lechene's Laboratory (30).

For correlative TEM/nanoSIMS, the samples were counterstained with uranyl acetate to detect stress granules. For the quantitative nanoSIMS measurements, samples were unstained to avoid a possible bias caused by uranyl ions.

To control that cellular RNA was actually labeled with^15^N-uridine, total RNA was extracted in NRK cells exposed to 100 μM ^15^N-uridine during 16 h using Trizol reagent and purified following manufacturer's recommendation (Quiagen, RNAeasy kit). Purified cellular RNA (final *A*_260_/*A*_280_ ratio = 1.9) was then deposited on carbon-coated electron microscopy grids for nanoSIMS analysis.

**Figure 1.**
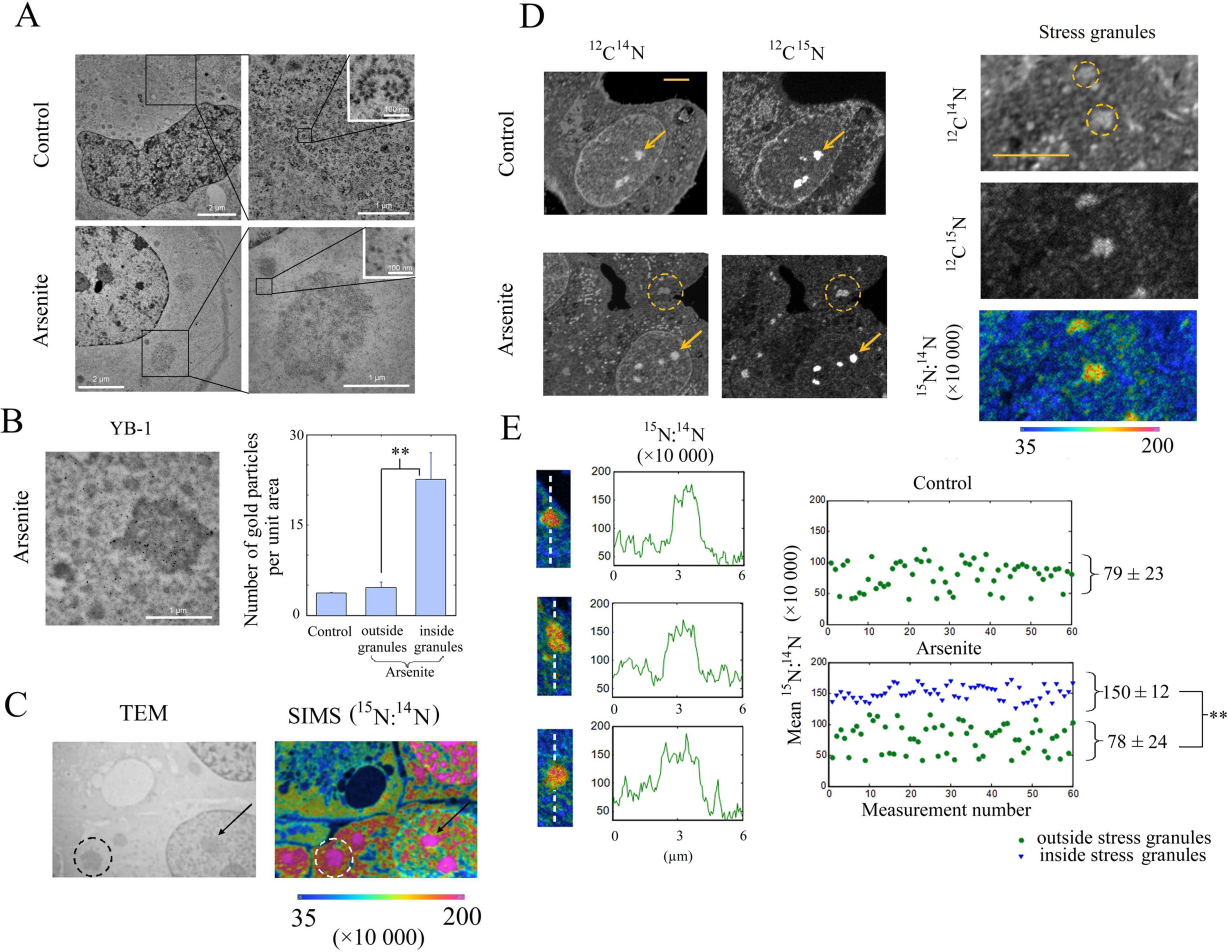
Stress granules in arsenite-treated cells are enriched in RNA compared to proteins. (**A**) NRK cells were untreated or exposed to 300 μM arsenite for 45 min. Transmission electron microscopy shows the presence of polysomes in control condition (higher magnification), which were not present after arsenite treatment. Instead, large and unstructured aggregates presumed to be stress granules appear in the cytoplasm. (**B**) Transmission electron microscopy imaging of immunogold anti-YB-1 labeled NRK cells shows a concentration of gold nanoparticles in the granules that appeared with arsenite. Statistical analysis of the quantification of the gold particle location in the cytoplasm of control and arsenite-treated cells and in the granules indicates an about 4-times enrichment of gold nanoparticles in the granules. Results are mean ± SD (*n* = 30). **P < 0.01 by *t*-test. (**C**) Correlative TEM/nanoSIMS microscopy of NRK cells treated with arsenite. The^15^N:^14^N ratio highlights the^15^N-enriched uridine-labeled RNA. Dashed circles and arrows point out a RNA-rich stress granule and a nucleolus, respectively. (**D**) nanoSIMS mapping of the ^12^C^14^N^−^ and ^12^C^15^N^−^ ion species and of the ^15^N:^14^N ratio in control and arsenite-treated cells (see ‘Materials and Methods’ section). In arsenite-treated cells, we observed the formation of stress granules (dashed circles) which can be distinguished both in the ^12^C^14^N^−^ (proteins and RNA) and the ^12^C^15^N^−^ (^15^N-uridine-labeled RNA) cartographies. As a control, arrows indicate the presence of RNA-rich nucleolus in both control and arsenite-treated cells. High magnification images of cytoplasmic stress granules show their enrichment in RNA compared to proteins as evidenced in the ^15^N:^14^N cartography. Scale bars: 5 μm. (**E**) Image gallery of stress granules and their respective line profile illustrating the increase of the^15^N:^14^N ratio in stress granules (mean value obtained over the 230 nm-thick dashed line). The scatter plots represent the mean ^15^N:^14^N ratio values measured in the cytoplasm of control and arsenite-treated cells (outside and inside stress granules in the latter case). The means were obtained by averaging the ratio over 0.5 μm^2^ areas. Statistical results are mean ± SD (*n* = 50). **P < 0.01 by *t-*test.

Mapping of the ^15^N:^14^N ratio showed an uniform distribution over a 9 μm^2^ area and statistical analysis of this ratio indicates that the RNA sample has a significant higher ^15^N content (^15^N:^14^N ratio = 3.96 ± 0.04%) compared to the cytoplasm of NRK cells exposed to ^15^N-uridine (^15^N:^14^N ratio = 0.79 ± 0.23%), see Figure 1E. This result thus indicates that ^15^N-uridine has been significantly incorporated to cellular RNA which is necessary to keep a high signal-to-noise ratio in the ^15^N:^14^N cartography.

### Atomic force microscopy

Human recombinant YB-1 was expressed in *Escherichia coli* and purified as previously described (26). Purified protein was dialyzed against 200 mM NaCl, 20 mM HEPES–KOH pH 7.6, 1 mM Dithiothreitol (DTT) and stored at −80°C. Human recombinant TIA-1 was purchased from Novus Biological and we controlled that the protein can bind and also aggregate mRNA *in vitro*. Interactions between TIA-1, YB-1 and mRNA were probed in 20 mM 2-(N-morpholino)ethanesulfonic acid (MES)–KOH, pH 6.8, 1 mM ethylene glycol tetraacetic acid (EGTA), 50 mM KCl, 2 mM MgCl_2_ and 10 μM spermidine. Ten microliters of each sample were deposited on freshly cleaved mica and dried for AFM imaging as described previously (31). All AFM experiments were performed in intermittent mode with a multimode AFM instrument (Digital Instruments, Veeco, Santa Barbara, CA, USA) operating with a Nanoscope IIIa controller. We used AC160TS silicon cantilevers (Olympus, Hamburg, Germany) with resonance frequencies of ∼300 kHz. The applied force was minimized as much as possible to reduce sample deformation. Images were collected at a scan frequency of 1.5 Hz.

### Cell culture

NRK-52E cells (ATCC, Manassas, VA, USA) are rat kidney epithelial cells originating from proximal tubules. They were cultured in Dulbecco's modified Eagle's medium (DMEM) supplemented with 5% (v/v) fetal bovine serum (FBS), 2 mM l-glutamine and 1% antibiotics (penicillin and streptomycin) in a humidified 5% CO_2_ atmosphere at 37°C.

### Immunofluorescence

NRK cells were fixed with 4% PFA, 150 mM sucrose in PBS for 20 min at 37°C. After fixation, cells were then washed and incubated for 12 h at 4°C with either rabbit anti-YB-1, mouse anti-HuR antibody (Molecular Probes, 10 μg/ml), mouse monoclonal anti-tubulin antibody E7 (1:2000 dilution), rabbit anti-phospho-eIF2α antibody (Cell Signaling, 1:2000 dilution), mouse anti-ubiquitin (abcam, ab7254, 1:1000 dilution), goat anti-vimentin (Santa Cruz, SC-7558, 1:1000 dilution) and goat anti-TIA-1 (Santa Cruz, SC-1751, 1:200 dilution). Cells were then washed extensively in PBS and incubated for 1 h with fluorochrome (Alexa Fluor^®^488 and 594)-coupled secondary antibodies (Invitrogen) in blocking solution. After final washes with PBS, samples were mounted for fluorescence microscopy analysis. The measurements of the mean GFP, anti-HuR or anti-TIA-1 fluorescence intensities were performed using the ImageJ software. The mean integrated intensity per area unit was measured in the specified locations (nucleus, cytoplasm). Integrated intensities of stress granules were defined as the intensity of the anti-YB-1 immunofluorescence signal integrated over the sum of the granule areas minus the integrated intensity in the surrounding cytoplasmic background.

### Immuno-blotting

NRK cells were washed once in PBS and lysed in 50 mM Tris–HCl (pH 7.5), 150 mM NaCl, 1% Nonidet P40, 1 mM EDTA in the presence of phosphatase inhibitors (2 mM orthovanadate, 25 mM NaF and 2 mM β-glycerophosphate) and a protease inhibitor cocktail (Roche). Lysates were centrifuged at 14 000 × *g* for 15 min at 4°C, and supernatants were collected. Proteins were separated by sodium dodecyl sulfate-polyacrylamide gel electrophoresis (SDS-PAGE) (12% acrylamide) and transferred onto a Polyvinylidene fluoride (PVDF) membrane (Invitrogen). The membranes were blocked in 5% (w/v) non-fat dried milk/PBS for 30 min at room temperature (20°C), and incubated for 12 h at 4°C with primary antibodies (anti-tubulin and anti-phospho-eIF2α). Bound antibodies were detected and quantified using anti-rabbit-IRDye 800 or anti-mouse-IRDye 680 secondary antibodies (Odyssey, 1:2000 dilution) with an Odyssey imaging system (LI-COR Biosciences).

### Cell enucleation procedure

NRK cells grown on 6 mm-diameter glass coverslips were treated with 1 μM cytochalasin D for 1 h in order to disrupt F-actin. Coverslips were then placed upside down in 1.5 ml eppendorf tubes, and centrifuged at 8000 × *g* for 25 min at 34°C. Under such conditions, we obtained on a same coverslip coexisting enucleated and non-enucleated cells. Samples were then washed with PBS and allowed to recover in the culture medium for 1 h.

### Transfection of NRK cells with YB-1 plasmid and small interfering RNA

The cDNA encoding the full-length YB-1 was cloned into the XhoI and BamH1 sites of the pEGFP-C3 vector (Clontech). The plasmid encoding the full-length human HuR (untagged) was a gift of Dr Sandrine Baghdoyan (ISTEM, Evry, France). Transfection of DNA plasmids in NRK cells was realized using lipofectamine™ 2000 (Invitrogen). In the case of small interfering RNA transfection, we used small interfering RNA (siRNA) specifically targeting YB-1 (CCACGCAAUUACCAGCAAAdTdT), HuR (Rn_Gja1-1; Qiagen) and negative control siRNA (AllStars Negative control siRNA, Qiagen) at a final concentration of 100 nM with the Hyperfect transfection reagent for 36 h according to the manufacturer's instructions.

### mRNA synthesis and preparation

The cDNA encoding α-globin containing a coding part, the sequence of 3′UTR and 50 nt poly(dA) was inserted into the NcoI and BamHI sites of the pET-28a vector (Novagen), resulting in the pET-28a-α-globin plasmid. The SP72-2Luc plasmid was obtained by cloning two full-length cDNAs of luciferases, separated by a polylinker, into the EcoRV and XbaI sites of the pSP72 vector (Promega). The pET-28a-α-globin and SP72-2Luc plasmids were used as templates for the synthesis by T7 polymerase of α-globin (660 nt) and 2Luc mRNAs (3000 nt), respectively. After transcription, unincorporated NTPs were removed by gel-filtration through a NAP-5 column (GE Healthcare) and mRNAs were further isolated with RNAble (Eurobio) following manufacturer's recommendation.

### mRNA and ssDNA, and dsDNA transfection

One microgram of mRNA (α-globin, and 2Luc mRNAs, fluorescent or not), ssDNA (M13, Invitrogen) or dsDNA (pUC19, Invitrogen) were incubated with 1 μl lipofectamine™ 2000 (Invitrogen) for 20 min in 100 μl of DMEM. NRK cells were then exposed to these preformed lipoplexes added to 1 ml of culture medium. Cells were fixed after transfection at indicated times for immuno-fluorescence analysis.

## RESULTS

### The RNA:protein ratio is significantly higher in stress granules than in the surrounding cytoplasm

The accumulation of RNA in stress granules was evidenced by combining TEM imaging with nanoSIMS. TEM was used to identify stress granules whereas nanoSIMS allowed us to measure the ^15^N-uridine labeled RNA content at a resolution below 100 nm (see ‘Materials and Methods’ section). Sodium arsenite (300 μM for 45 min), which causes a severe oxidative stress, was used to induce the formation of micrometric stress granules. In control NRK cells, polysomes were clearly visible in TEM images. In arsenite-stressed cells, as expected and in agreement with previous reports (12,32), they were absent (Figure 1A) due to the inhibition of translation initiation. In parallel, micrometric granular aggregates considered as stress granules appeared in the cytoplasm (Figure 1A and B). We confirmed that these granular aggregates were actual stress granules by imaging the endogenous YB-1 protein by TEM via immunogold labeling (YB-1, a RNA-binding protein, is a good marker of stress granules (33)). YB-1 was found about 4-times enriched in the micrometric granular aggregates that appeared in arsenite-treated cells compared to the surrounding cytoplasm (Figure 1B).

NanoSIMS was then used to detect ^15^N-uridine labeled RNA molecules. We first controlled that the ^15^N:^14^N ratio measured in RNA extracted from NRK cells exposed to ^15^N-uridine for 16 h was significantly larger than the natural ^15^N:^14^N ratio (3.96 ± 0.04% and 0.36%, respectively, see ‘Materials and Methods’ section). We then measured the ^15^N:^14^N ratio across thin sections prepared from NRK cells. The analyses of control cells showed that the ^15^N:^14^N ratio in the cytoplasm oscillates from about 0.4%, in regions of polysome exclusion such as the Golgi apparatus, to ∼1%, in regions most probably corresponding to membrane-bound polysomes (Figure 1C and D). Interestingly, in arsenite-treated NRK cells, the ^15^N:^14^N ratio could rise up to 1.6% in stress granules which could then be easily distinguished from the surrounding cytoplasm (Figure 1D and E). In addition, we noticed a stronger nanoSIMS signal of the ^12^C^14^N^−^ ions in stress granules than in the rest of the cytoplasm (Figure 1D) due to ^14^N atoms coming from both proteins (unlabeled) and RNA (non-^15^N-uridine labeled nucleotides) that have accumulated in stress granules. Consequently, the elevated ^15^N:^14^N ratio indicates that proportionally more RNAs than proteins accumulated in stress granules compared to the surrounding cytoplasm. If the accumulation of proteins had been relatively higher than that of RNA in stress granules, we would have observed a lower ^15^N:^14^N ratio in stress granules than in the surrounding cytoplasm, despite a net RNA accumulation.

The relative excess of RNA compared to proteins in stress granules is however not a proof that an RNA excess plays a key role in stress granule assembly but prompted us to further consider this issue. In addition, as all RNA (rRNA, tRNA and mRNA) were labeled during the 16 h incubation period with ^15^N-uridine, we cannot determine the relative fraction of these RNAs in stress granules. So far, mRNA and the 40S ribosomal subunit but not the 80S subunit were found in stress granules (7,32) and thus mostly participate in the RNA enrichment observed here. In line with this, we controlled that both mRNA and the 40S subunit are indeed enriched in stress granules in contrast to the 80S subunit (Supplementary Figure S1A and B).

Even though both the 40S ribosomal subunit and mRNA can participate in stress granule assembly, the prion-like proteins that promote stress granule assembly like TIA-1 and G3BP are mostly mRNA-binding proteins rather than ribosomal proteins, which argue for a critical role of mRNA in stress granule assembly. In addition, as previously estimated ((32), Supplementary Figure S1B), the relative accumulation of mRNA in stress granules was higher than that of the 40S subunit. We then chose to focus our attention on the contribution of nonpolysomal mRNA to stress granule dynamics rather than on ribosomal RNA.

### Nonpolysomal mRNA is required for stress granule assembly after short-term inhibition of proteasome or HSP70 chaperone activities

**Figure 2.**
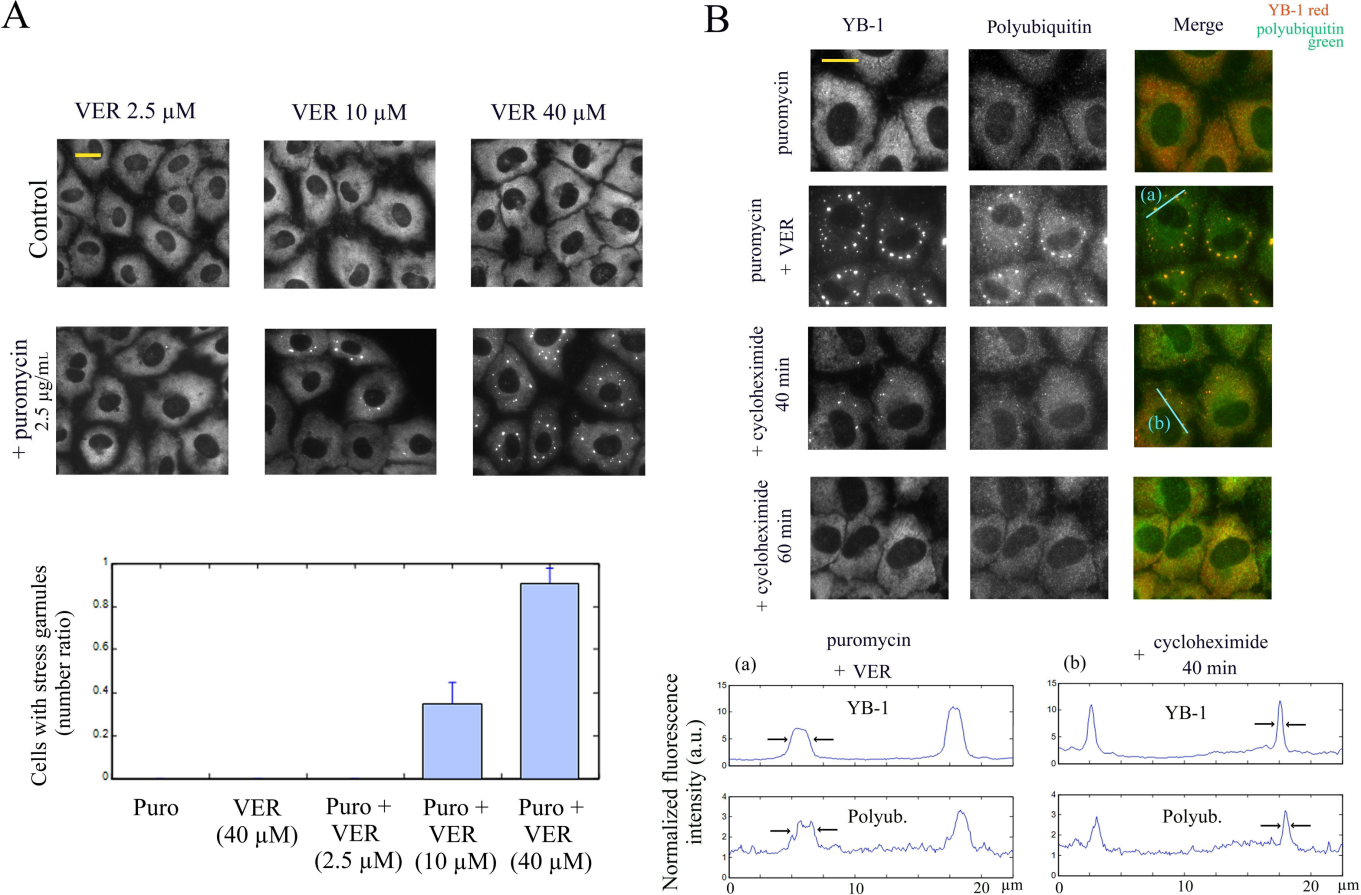
Nonpolysomal mRNA is required for the maintenance of polyubiquitin-rich protein aggregates that co-localize with stress granules after HSP70 inhibition and puromycin treatment. (**A**) NRK cells were treated for 3 h, as indicated, and labeled with anti-YB1 antibody. Puromycin leads to the appearance of cytoplasmic stress granules in cells treated with VER-155008 (VER). Statistical results are mean ± SD obtained on three different samples. Puro, puromycin (2.5 μg/ml). (**B**) NRK cells were exposed to 10 μM VER-155008 in combination with 2.5 μg/ml puromycin for 3 h and then to 20 μg/ml cycloheximide in the continuous presence of VER-155008 and puromycin, as indicated. Anti-polyubiquitin labeling was used to reveal the presence of polyubiquitinated proteins in stress granules. Line profiles of the anti-YB-1 and anti-polyubiquitin fluorescence intensities show the co-localization of stress granules and polyubiquitinated-protein aggregates and their progressive dissociation upon cycloheximide exposure. No polyubiquitinated protein aggregates were observed in the absence of stress granules after cycloheximide treatment for 1 h. Scale bar: 15 μm.

The key role of nonpolysomal mRNA in stress granules has been known since cycloheximide and emetine, which prevent polysome dissociation, were shown to inhibit stress granule assembly (21). On the other hand, puromycin, which dissociates polysomes, promotes stress granule assembly but is not sufficient by itself to trigger massive stress granule assembly, even at high concentration ((10 μg/ml), (21)). In line with this, at the concentration used herein to treat NRK cells (2.5 μg/ml), puromycin treatment is not sufficient to induce stress granules. The assembly of stress granules could then result from the concomitant presence of nonpolysomal mRNA and pro-aggregating factors. Among the pro-aggregating factors are misfolded proteins that appear during stress and have the tendency to form aggregates like aggresomes (34). Using inhibitors of heat shock protein chaperone (VER-155008 (35) and quercetin (36)), we manipulated both nonpolysomal mRNA or misfolded protein levels in the cytoplasm and analyzed the impact of such manipulations on stress granule formation. VER-155008 and quercetin do not lead to the appearance of stress granules by themselves under the conditions tested (Figure 2A and Supplementary Figure S2A). However, when puromycin was combined to VER-155008 or quercetin, stress granules appeared and persisted for hours in the cytoplasm (Figure 2A and Supplementary Figure S2A and video 1). This shows that both polysome dissociation and protein misfolding contribute to stress granule assembly. Similar observations were made after MG132 treatment which inhibits proteasome activity when translation initiation was blocked ((37) and Supplementary Figure S3).

Using an anti-polyubuquitin antibody as marker of protein aggregates, we then investigated the putative role of protein aggregates as initiators of stress granule formation as they were reported to co-localize with stress granules (38). Under our conditions, protein aggregates were, as expected, found in the stress granules formed after exposure to puromycin combined to VER-155008 or MG132 (Figure 2B and Supplementary Figure S2B). This may suggest an implication of protein aggregates in the nucleation of stress granules as misfolded proteins could have first formed aggregates and subsequently attracted nonpolysomal mRNA in the protein aggregates. To test this hypothesis, we examined whether protein aggregates could persist after the re-loading of ribosomes on mRNA via cycloheximide treatment leading to the dissociation of stress granules (video 2). The results show a clear dispersal of the protein aggregates upon the dissociation of preformed stress granules (Figure 2B), which indicates that the aggregation of misfolded protein depends on the presence of mRNA under the tested conditions. Altogether, the results suggest that the presence of nonpolysomal mRNA is necessary to form protein aggregates when the HSP70 or proteasome activities are inhibited.

### mRNA or ssDNA delivery to the cytoplasm triggers stress granule assembly in puromycin-treated cells

**Figure 3.**
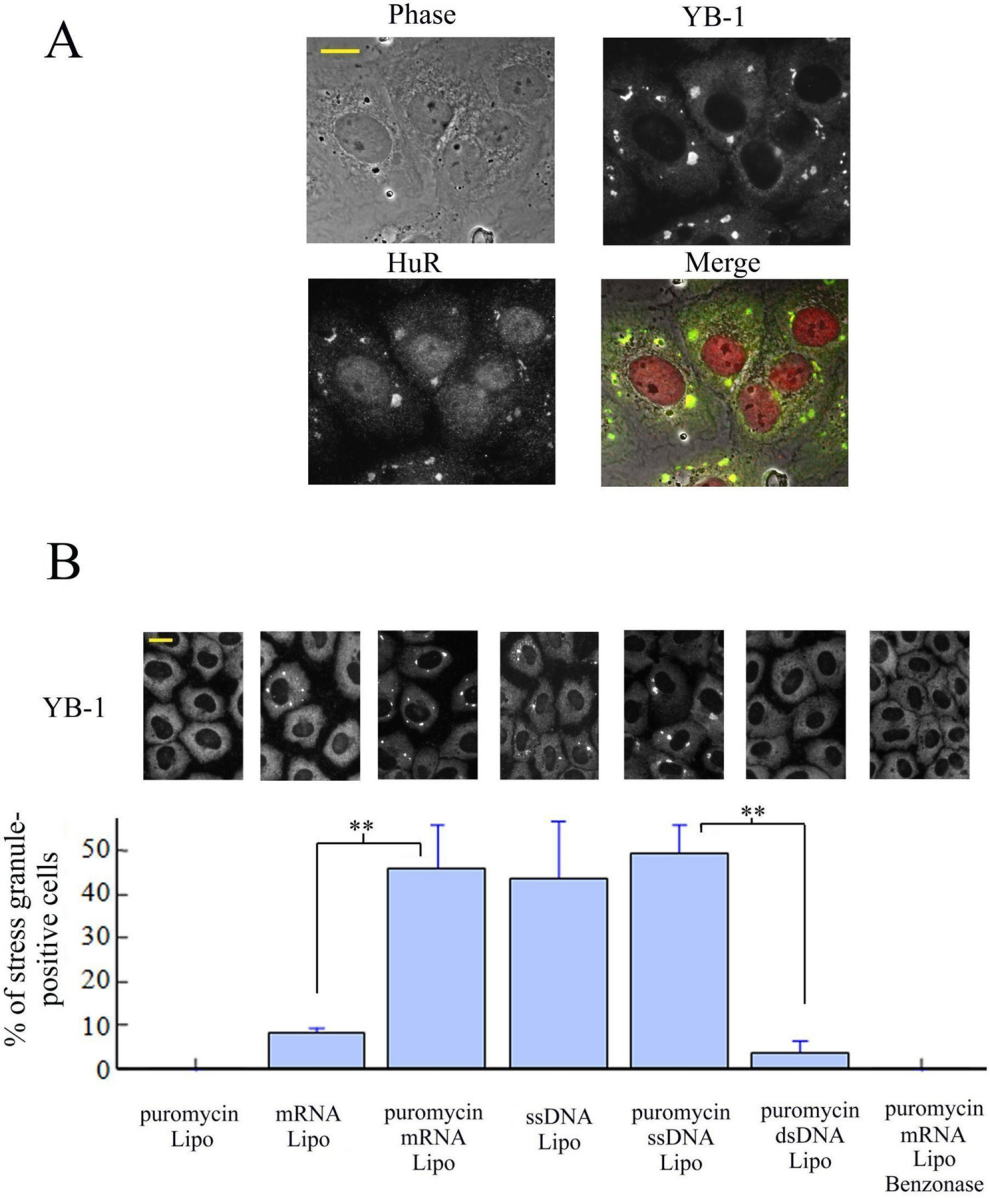
mRNA or ssDNA but not dsDNA transfections lead to stress granule assembly in puromycin-treated cells. (**A**) 1 μg of 2Luc mRNA synthesized *in vitro* was transfected in NRK cells using lipofectamine in the presence of 2.5 μg/ml puromycin (see ‘Materials and Methods’ section). Anti-HuR and anti-YB-1 labeling show the formation of stress granules 3 h after transfection. (**B**) NRK cells were transfected using lipofectamine (Lipo) with 1 μg of α-globin mRNA, M13 ssDNA, or linearized pUC19 dsDNA in the presence or absence of puromycin for 3 h. Benzonase treatment was performed before the formation of lipoplexes. The statistical analysis was obtained on three different samples. Results are mean ± SD (*n* = 3). Both mRNA and ssDNA lead to a significant formation of stress granules in puromycin-treated cells. Anti-YB-1 labeling shows the formation of stress granules 3 h after transfection.

To further explore the role of nonpolysomal RNA on stress granule assembly, we increased the local concentration of mRNA by transfection of α-globin and 2Luc mRNAs synthesized *in vitro* using cationic liposomes as vectors. In puromycin-treated cells, stress granules appeared in the cytoplasm 90 min after the start of transfection of both α-globin or 2Luc mRNAs (Figure 3A and Supplementary Figure S4). The intensity of the fluorescence of stress granules positively correlated with the amount of transfected mRNA (Supplementary Figure S4). We found that in the absence of puromycin, transfected mRNAs rarely induce stress granules (Figure 3B) which suggests that puromycin most probably also prevents the loading of ribosomes onto exogenous mRNA. Interestingly, we observed that single-stranded DNA (ssDNA) induces stress granules both in the presence or absence of puromycin, in agreement with the fact that ssDNA cannot load ribosomes. On the other hand, double-stranded DNA (dsDNA) failed to induce stress granule assembly. Stress granule assembly appears then to require the presence of long single-stranded polynucleotides free of ribosomes without specificity for RNA or DNA which agrees with the fact that many RNA-binding proteins have a strong affinity for both mRNA and ssDNA. To strengthen this finding, we controlled that stress granule assembly did not occur when mRNA was digested by benzonase prior to its incorporation into the transfection reagent (Figure 3B).

### Cytoplasmic YB-1 prevents mRNA granule assembly in cells and *in vitro*

If an increase of nonpolysomal mRNA concentration is actually the primary event for protein self-attraction and subsequent formation of stress granule assembly, we should prevent the formation of stress granules by limiting the access of aggregation-prone proteins like TIA-1, G3BP and (or) misfolded proteins to nonpolysomal mRNA. To test this hypothesis, we chose YB-1, a cytoplasmic mRNA-binding protein (28) which could directly prevent mRNA aggregation for the following reasons: (i) YB-1 has a higher affinity for nonpolysomal than polysomal mRNA (39) so that it can then effectively compete for the binding to nonpolysomal mRNA with other RNA-binding proteins; (ii) YB-1 is dispensable for stress granule assembly as its silencing does not impede stress granule assembly (Supplementary Figure S5A); (iii) YB-1 is known to form stable isolated mRNPs (28). Importantly, YB-1 overexpression in cells could prevent stress granule assembly (40), though an increased HSP70 activity was put forward to explain such behavior.

**Figure 4.**
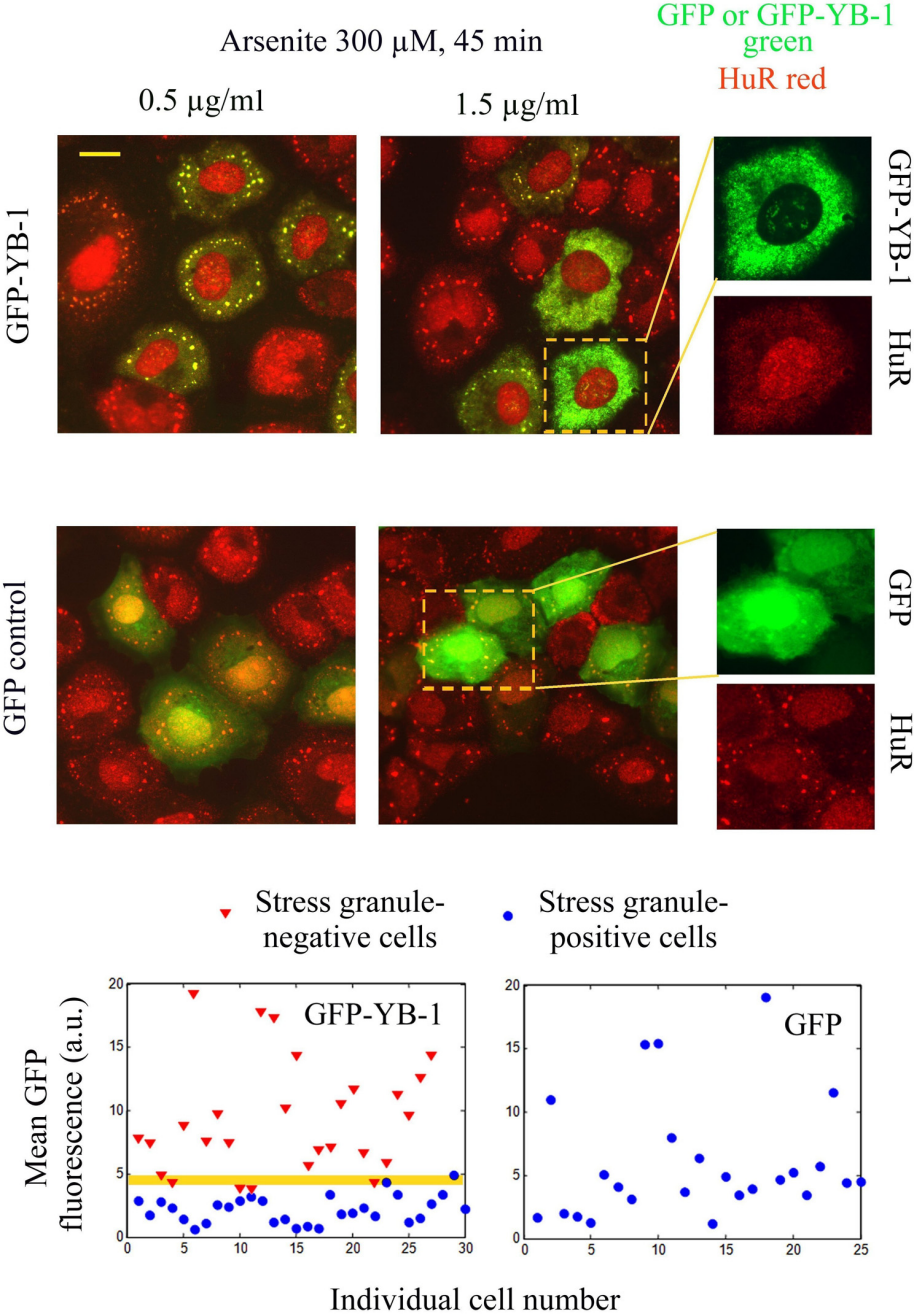
YB-1 inhibits stress granule assembly above a critical expression level. GFP-YB-1 or GFP-transfected NRK cells were exposed to 300 μM arsenite for 45 min. The amount of plasmid used is indicated above the pictures. Higher magnification images show that high expression levels of GFP-YB-1 (but not of GFP) inhibit stress granule assembly. Scatter plots of the mean GFP-YB-1 or GFP cytoplasmic fluorescence show that, above a critical expression level of GFP-YB-1, stress granule assembly is impaired. Such a pattern is not observed for GFP expression alone. The null hypothesis that the GFP-YB-1 fluorescence intensities of stress granule positive and negative cells are similar for the two populations displayed in the scatter plot is rejected at the 5% significance level (*t*-test).

**Figure 5.**
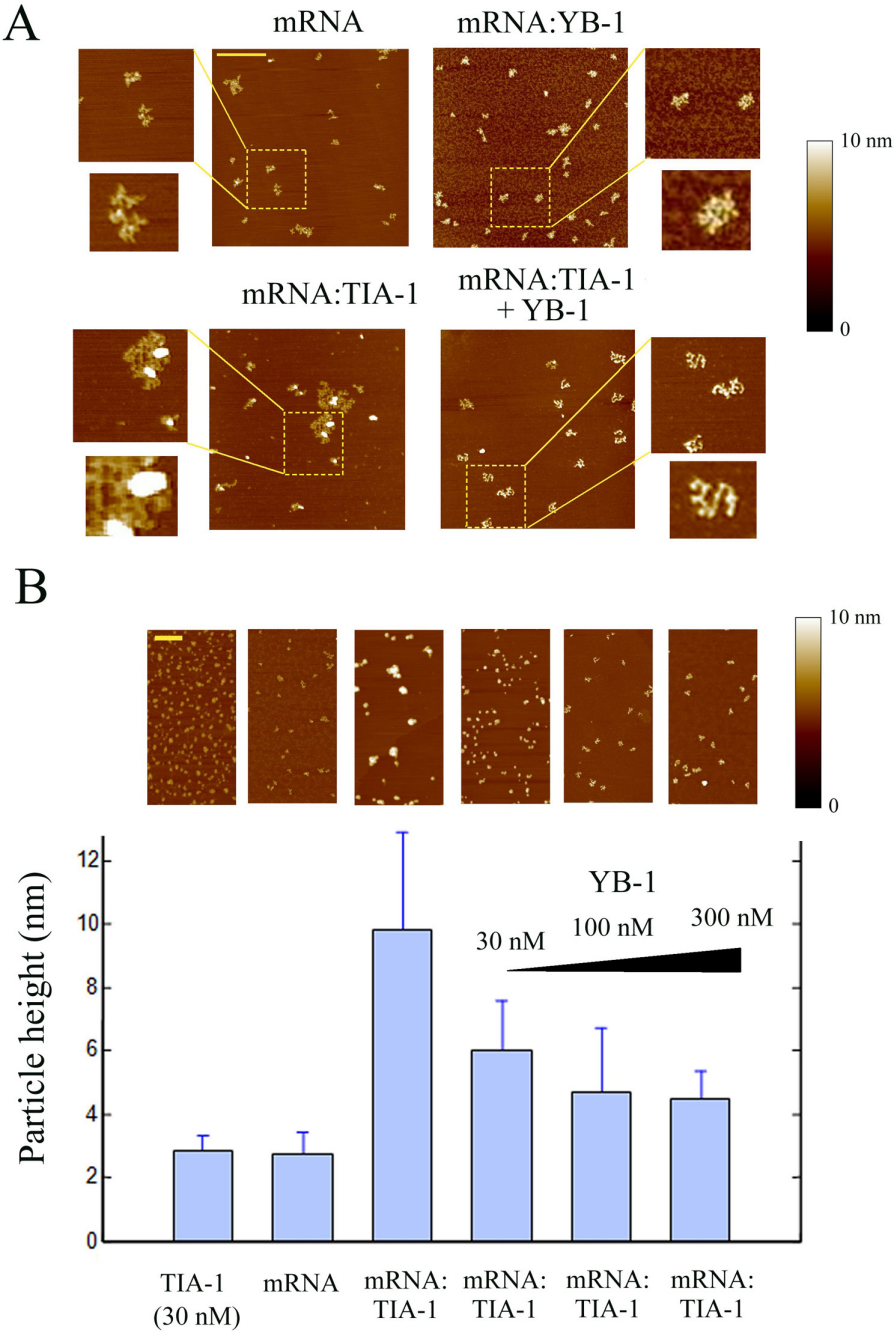
YB-1 dissociates RNA granules *in vitro* as revealed by atomic force microscopy. (**A**) As control, naked 2Luc mRNA (2 μg/ml) deposited on a mica surface was clearly detected by AFM. In the presence of 100 nM YB-1, single isolated mRNPs were detected. On the other hand, in the presence of 15 nM TIA-1, mRNA:TIA-1 complexes form aggregates. Interestingly, when 100 nM YB-1 was added to preformed mRNA:TIA-1 aggregates for 5 min, a clear dissociation of RNA granules into isolated mRNP was observed. Scale bar: 0.5 μm. (**B**) Statistical analyses of the particle height on the mica surface. TIA-1 (30 nM) in the absence of RNA is attracted on the negatively-charged surface and tends to form small TIA-1 aggregates (2.8 ± 0.5 nm), as expected from its self-attracting domain. In the presence of 2Luc mRNA (2 μg/ml), we noticed the appearance of large mRNA:TIA-1 aggregates (9.8 ± 3.1 nm). These large aggregates consequently contained both mRNA and TIA-1. Addition of increasing concentration of YB-1 progressively dislocates the mRNA:TIA-1 aggregates and leads to the appearance of isolated mRNPs after 5 min. Scale bar: 0.5 μm. The ‘particle analysis’ application of the nanoscope IIIa software (version 5) over at least 200 particles was used to provide mean heights and standard deviations.

We first controlled whether YB-1 in our conditions behaves as a negative regulator of stress granule assembly, as previously described (40). We found that, while low expression levels of GFP-YB-1 do not interfere with arsenite-induced stress granules, above a threshold, stress granule assembly is significantly inhibited (Figure 4). In addition, arguing against a role of HSP70 (40), we observed that high level of YB-1 similarly leads to the inhibition of stress granule assembly when both the HSP70 activity and its expression were inhibited by VER-155008 plus puromycin treatment (Supplementary Figure S5B). The inhibitory effect of YB-1 expression on stress granule assembly could then rather result from the direct binding of YB-1 to mRNA. In order to address this issue, we analyzed the effect of YB-1 on mRNA:TIA-1 aggregates *in vitro* by AFM. This technique allows the visualization at a nanometric resolution of protein/nucleic acid complexes (‘Materials and Methods’ section, (41)). By mixing 2Luc mRNA and YB-1, we detected the presence of isolated mRNPs with a beads-on-a-string structure, as previously found by AFM (Figure 5A, (28)). They were well dispersed and isolated from each other. In contrast, TIA-1 in the presence of 2Luc mRNA leads to the formation of mRNP aggregates (Figure 5A and B), as expected from its prion-like self-attracting domain. Upon the addition of YB-1 to the mRNA:TIA-1 aggregates, we then clearly observed their dissociation into isolated mRNPs. YB-1 is then able to directly dissociate mRNA granules *in vitro* and, by a similar mechanism, potentially inhibits stress granule assembly when overexpressed in cells.

### Translocation of nuclear RNA-binding proteins from the nucleus to the cytoplasm is dispensable for stress granule assembly

**Figure 6.**
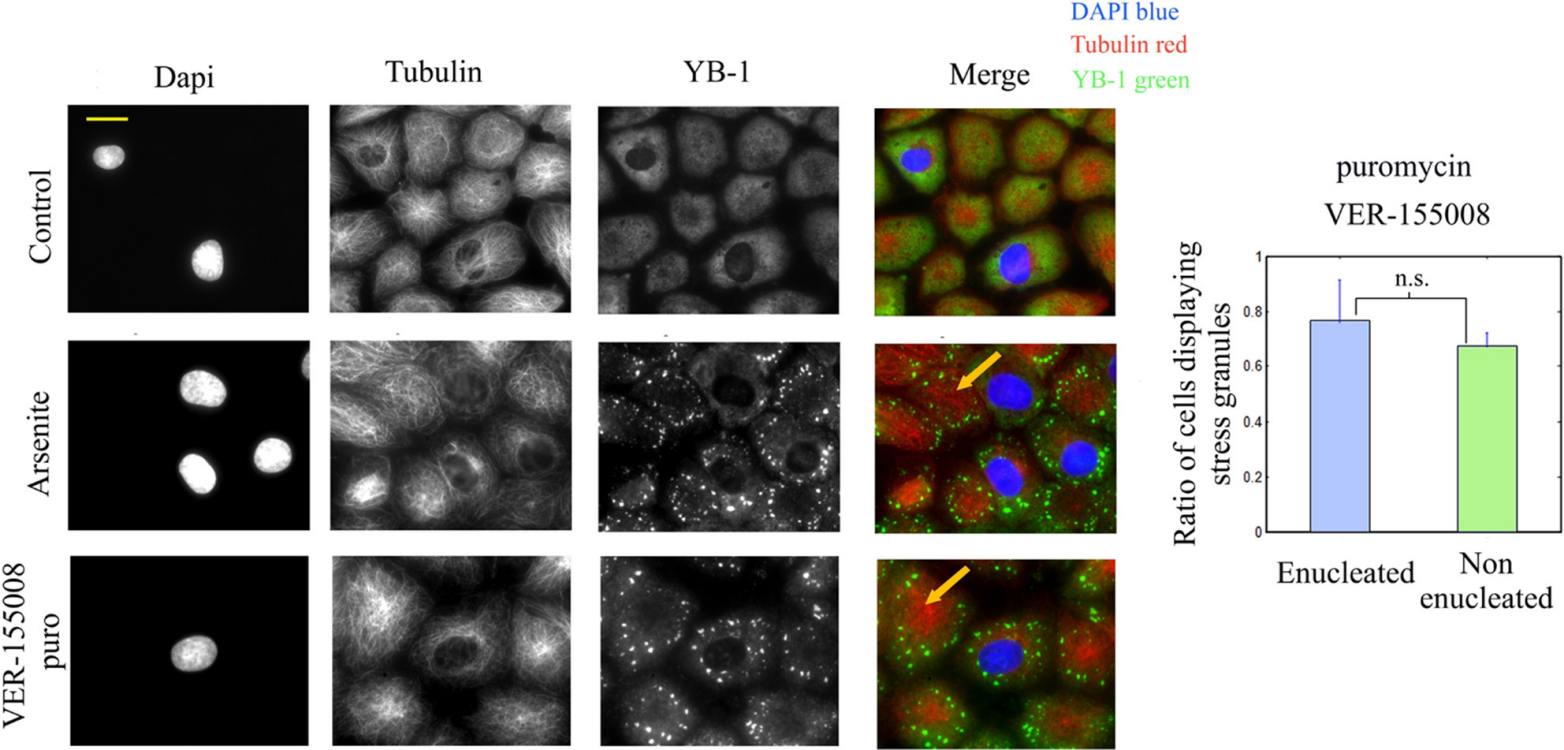
Enucleated NRK cells do form stress granules. Mix populations of enucleated and non-enucleated NRK cells were exposed to 300 μM arsenite for 45 min or 10 μM VER-155008 plus 2.5 μg/ml puromycin for 90 min. Cells were then stained to observe the nuclei (DAPI), microtubules (anti-tubulin) and stress granules (anti-YB-1). As indicated by arrows, enucleated cells clearly display stress granules. The statistical analysis shows that the percentage of stress-granule positive cells is similar in both enucleated and non-enucleated cells after VER-155008 plus puromycin exposure. Results are mean ± SD obtained on three different samples. n.s., not significant by *t-*test.

We investigated whether the shuttling of RNA-binding proteins like TIA-1 from the nucleus to the cytoplasm is necessary for stress granule assembly. To that end, we analyzed the ability of enucleated cells to form stress granules. Indeed, polysomes are known to be present in enucleated cells and protein synthesis is still going on unabated for at least 90 min after enucleation (42). Enucleation of NRK cells was realized by centrifugation of cells previously treated with cytochalasin D to disrupt the F-actin network. The experimental protocol was further adjusted to obtain the enucleation of only a fraction of the cell population in order to compare the behavior of enucleated and non-enucleated cells exposed to similar conditions (see ‘Materials and Methods’ section). After centrifugation, cytochalasin D was washed out and cells were allowed to recover for 1 h. Under such conditions, the F-actin network reformed and both enucleated and non-enucleated cells recovered a normal morphology. We checked that the microtubule network was also unaffected (Figure 6), since it is a critical parameter for micrometric stress granule assembly (43). In addition, cytochalasin D does not bias the shuttling of mRNA-binding proteins like TIA-1 or HuR (Supplementary Figure S6A) and similar cytoplasmic levels for HuR and TIA-1 were found in enucleated and non-enucleated cells (Supplementary Figure S6B and C).

Interestingly, after arsenite or puromycin/VER-155008 exposure, both enucleated and non- enucleated cells retain their ability to form stress granules (Figure 6). In enucleated cells, the distribution of stress granules appeared inhomogeneous with a marked avoidance of the cell center, most probably due to the star-like distribution of microtubules (Supplementary Figure S7A). To ensure that the observed granules in enucleated cells were actual stress granules, the presence of mRNA was observed in these granules via *in situ* hybridization (Supplementary Figure S6D). We then also examined whether stress granules formed before the enucleation process persisted after enucleation to determine whether nuclear factors are needed for their maintenance. The results showed that both enucleated and non-enucleated cells displayed stress granules 1 h after enucleation in puromycin/VER-155008 treated cells (Supplementary Figure S7B). The shuttling of nuclear proteins to the cytoplasm seems not necessary for both stress granule assembly and their persistence. The proteins present in the cytoplast, including cytoplasmic TIA-1, are thus sufficient to trigger stress granule assembly.

### Translocation of nuclear RNA-binding proteins could inhibit stress granule assembly

After the demonstration that the translocation of nuclear RNA-binding proteins is dispensable for stress granule assembly, we examined whether such proteins could rather decrease the accessibility of aggregation-prone proteins to nonpolysomal mRNA during stress and thus contribute to the inhibition of stress granule assembly. To that end, we used Actinomycin D (ActD), an inhibitor of transcription, which induces the translocation of nuclear HuR (44) and TIA-1 (18) to the cytoplasm, most probably along with other RNA-binding proteins. We chose HuR as an example of shuttling mRNA-binding proteins that might not promote stress granule assembly, as expected from its structure which displays three RNA-binding motifs (RRM, (45)) but no clearly identified self-attracting domain. In line with this, overexpressing HuR does not induce per se stress granules ((46), Supplementary Figure S8A) while its silencing has no remarkable effect on stress granule assembly (Supplementary Figure S8B). HuR overexpression also tends to inhibit stress granule assembly in arsenite-stressed cells (Supplementary Figure S8C), although less clearly than YB-1 (Figure 4B). However, as HuR modifies the expression of other mRNA-binding proteins and positively regulates that of TIA-1 (47,48), the role HuR in stress granule assembly should then be interpreted with caution. The present results, however, indicate that HuR is most probably not a pro-aggregation factor.

**Figure 7.**
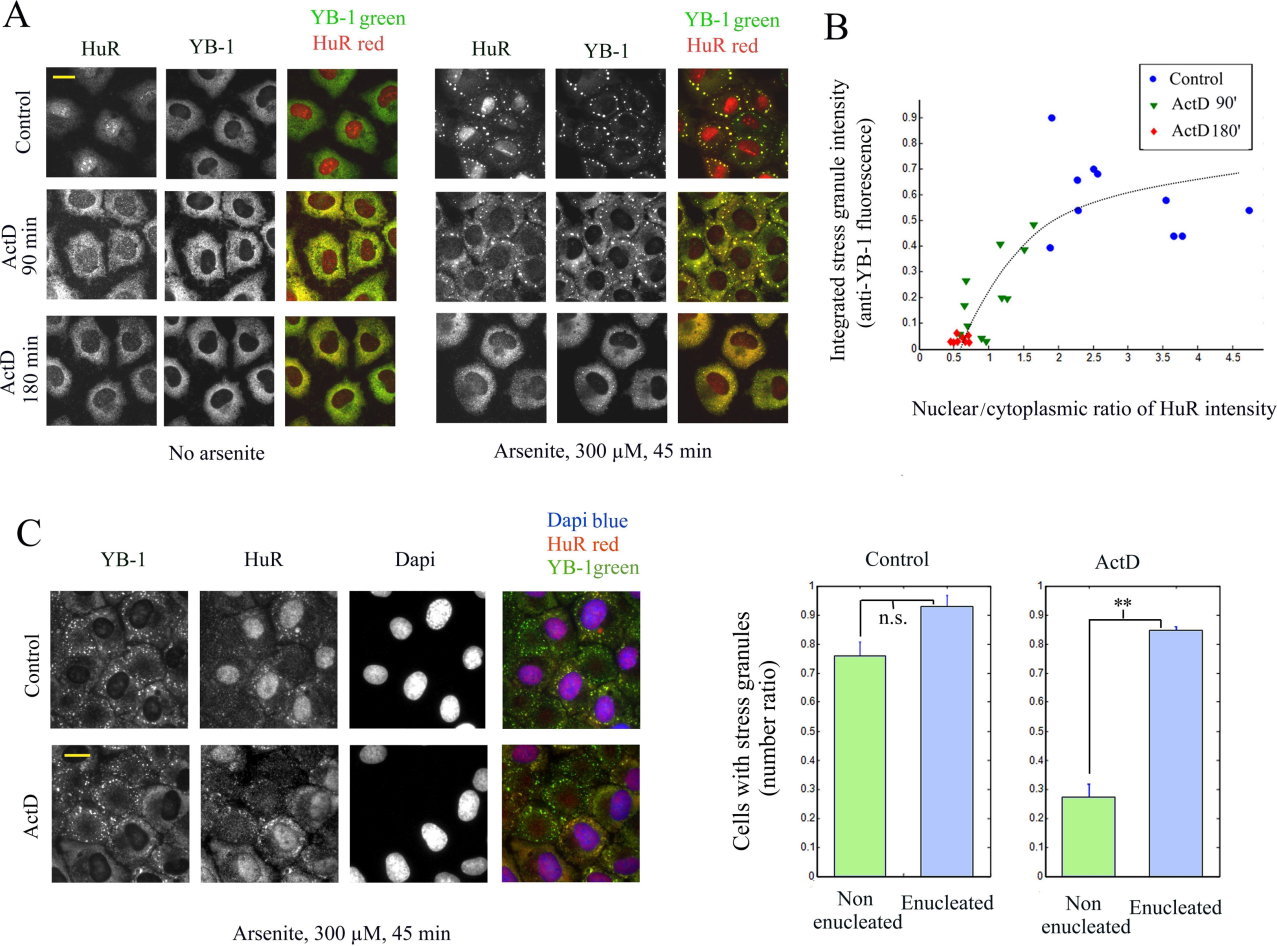
Actinomycin D prevents stress granule assembly and induces the translocation of nuclear HuR to the cytoplasm. (**A**) HuR translocation to the cytoplasm depends on the length of ActD treatment (5 μg/ml) and impacts stress granule formation under arsenite exposure (300 μM, 45 min). (**B**) Scatter plot of the integrated intensity of stress granules per cell (anti-YB1 staining) versus the nuclear–cytoplasmic ratio of HuR (mean anti-HuR fluorescence). The plot reveals a negative correlation between the formation of stress granules and the effectiveness of the HuR translocation to the cytoplasm. (**C**) Enucleated and non-enucleated cells were exposed to ActD prior to and during arsenite exposure (300 μM, 45 min). The graph shows the statistical measurement of the ratio of NRK cells displaying stress granules after arsenite exposure for both enucleated and non-enucleated cells with or without ActD. ActD leads to the inhibition of stress granule assembly only in non-enucleated cells. n.s., not significant; **P < 0.01 by *t*-test (three samples).

We pretreated cells with ActD for 90 and 180 min and then added either puromycin/VER-155008 or arsenite (Figure 7A). Interestingly, in both conditions, stress granules did not form despite the parallel increase of TIA-1 concentration in the cytoplasm (Supplementary Figure S8D). Furthermore, by varying the length of the ActD pretreatment, we were able to find a negative correlation between the cytoplasmic levels of HuR with the integrated fluorescence intensity of stress granules (Figure 7B). In control experiments, we checked that the mRNA cytoplasmic level was not significantly reduced during short term transcription inhibition using *in situ* hybridization (Supplementary Figure S9A). In addition, the phosphorylation of eIF2a, which induces polysome dissociation, is still efficient after ActD pre-treatment (Supplementary Figure S9B). To ascertain that the effect of ActD on stress granules is possibly due to the translocation of RNA-binding proteins to the cytoplasm and not to a direct interference with cytoplasmic RNA or its protein partners, we then analyzed its impact on the formation of stress granules in enucleated and non-enucleated cells. The results clearly show that ActD inhibits stress granule assembly only in non-enucleated cells but fails to do so in enucleated cells (Figure 7C).

**Figure 8.**
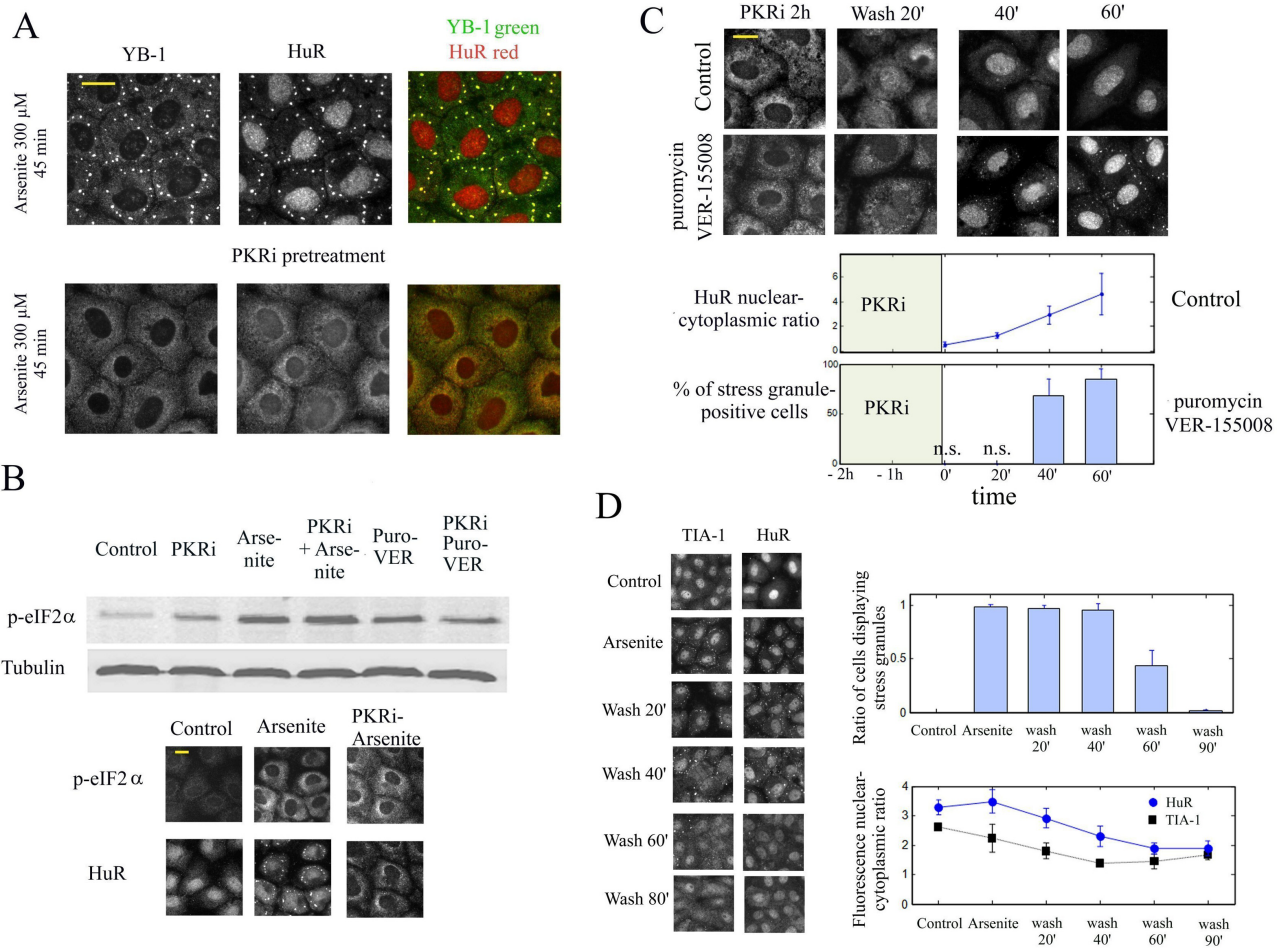
PKRi induces the translocation of nuclear HuR and reversibly inhibits stress granule assembly independently of the eIF2α phosphorylation level. (**A**) NRK cells were treated or not with 5 μM PKRi for 1 h prior to and during arsenite exposure (300 μM, 45 min). PKRi-pretreated cells failed to form stress granules and display a pronounced cytoplasmic HuR labeling compared to non-pretreated cells. (**B**) Representative western blot showing that a PKRi pretreatment as performed in (A) does not reduces the eIF2α phosphorylation level after both arsenite (300 μM, 45 min) or puromycin (2.5 μg/mL)/VER-155008 (10 μM) exposure. As a control, NRK cells were pretreated or not with 5 μM PKRi, exposed to arsenite and immuno-stained with anti-HuR and anti-*p*-eIF2α (phosphorylated form). (**C**) NRK cells were exposed to 5 μM PKRi for 1 h prior to and during puromycin (2.5 μg/ml)/VER-155008 (10 μM) exposure. PKRi was then removed from the incubation buffer. Under the continuous exposure to puromycin/VER-155008, YB-1 labeling reveals the progressive formation of stress granules with time. Statistical analyzes illustrate that the mean nuclear–cytoplasmic ratio of HuR intensities (anti-HUR staining) increases with time along with the percentage of stress-granule positive cells upon PKRi removal. Results are mean ± SD (*n* = 3). n.s., no significant appearance of stress granules. (**D**) After arsenite exposure (200 μM for 45 min), cells were allowed to recover in the absence of arsenite and then immuno-stained (anti-HuR and anti-YB-1 or anti-TIA-1). Statistical analyzes show the mean nuclear–cytoplasmic ratios of HuR and TIA-1 fluorescence intensities. Arsenite-preconditioning leads to the translocation of nuclear HuR and TIA-1. Results are mean ± SD over five different samples. *P < 0.05; **P < 0.01; by *t*-test.

To further evaluate the role of cytoplasmic translocation of nuclear RNA-binding proteins in stress granule regulation by a different mean, we screened the effects of other drugs that may generate the translocation of nuclear RNA-binding proteins, notably phosphatase or kinase inhibitors. The results of this screen show a remarkable effect of PKRi, a putative inhibitor of PKR kinase (49) and of other kinases (50) (Supplementary Figure S10). As PKRi induces a rapid and reversible translocation of both HuR and TIA-1 in NRK cells, this condition is particularly relevant to test our hypothesis (Figure 8A and Supplementary Figure S11A). We found that 1 h pretreatment of cells by PKRi combined with its administration during stress impede stress granule assembly by both arsenite or puromycin/VER-155008 treatments. To check whether PKRi inhibits stress granule assembly by preventing eIF2α phosphorylation, we analyzed the phosphorylation level of eIF2α in PKRi-treated cells after either arsenite or and puromycin/VER-155008 exposure. The results show that PKRi has no effect on eIF2α phosphorylation (Figure 8B) under the tested conditions, even though it may also change the phosphorylation level of RNA-binding proteins. Interestingly, under constant exposure to puromycin/VER-155008 combination, PKRi removal during stress led to the progressive reappearance of the stress granules in cells by 1 h (Figure 8C) along with the simultaneous recovery of nuclear HuR. So far, we observed that the translocation of HuR and TIA-1 induced by two different drugs, ActD and PKRi, results in stress granule dissociation. In absence of drug, cells could use a similar translocation strategy to dissociate stress granules, a necessary step to recover a normal translation after stress. To investigate such possibility, cells were exposed to arsenite for a limited period of time and then allowed to recover from arsenite stress for 90 min. Interestingly, we observed that the cytoplasmic levels of HuR and TIA-1 increased progressively after stress, which matched the progressive disappearance of stress granules (Figure 8D). In addition, we noticed that after the recovery period following the initial arsenite stress, another oxidative stress episode failed to trigger stress granule assembly (Supplementary Figure S11B) which suggests a persistence of ‘anti stress granule factors’ in the cytoplasm.

## DISCUSSION

**Figure 9.**
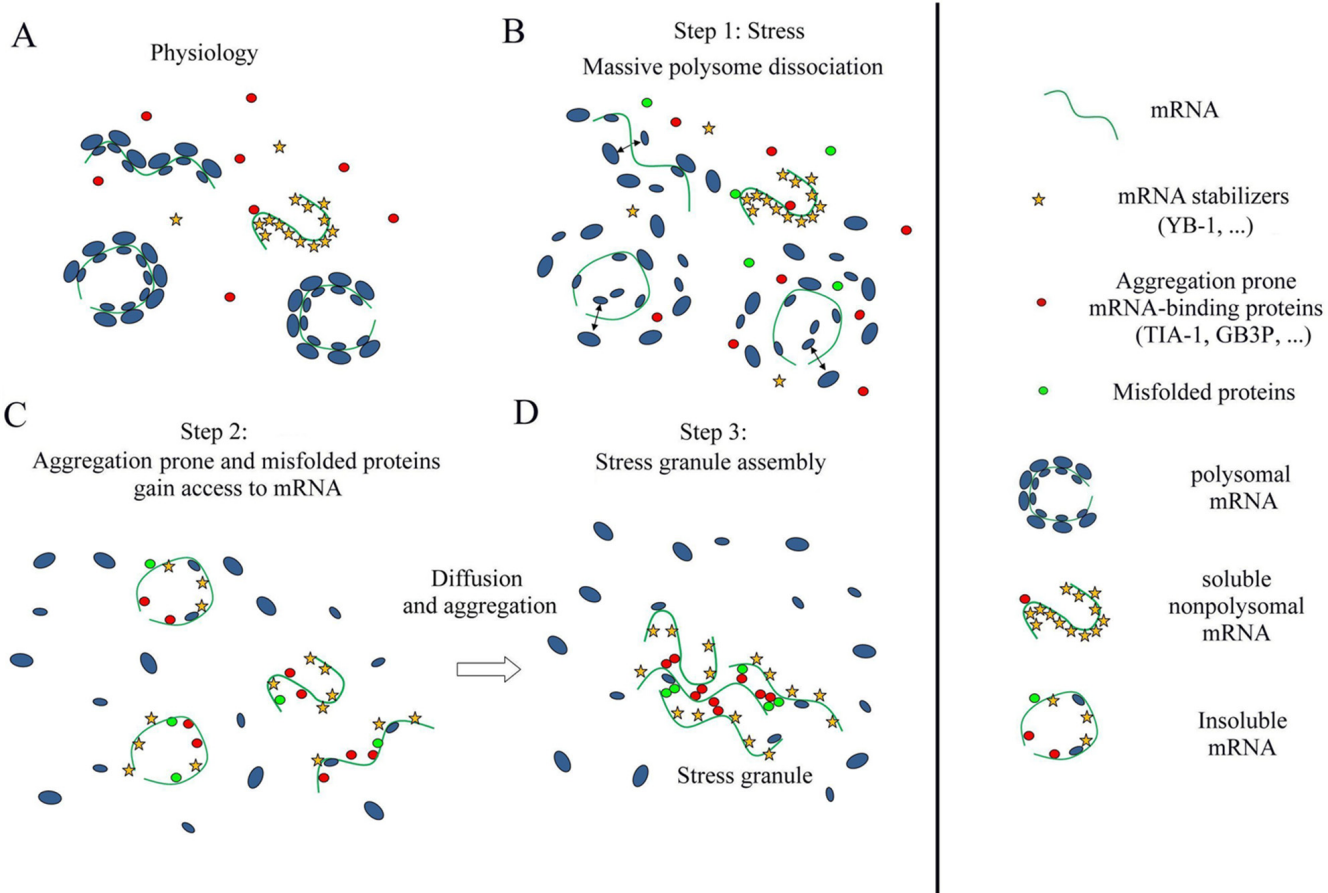
Schematic view of mRNA homeostasis and its deregulation during stress leading to stress granule assembly. (**A**) Polysomal mRNA coexists with soluble nonpolysomal RNA. Nonpolysomal mRNA is protected from aggregation via the binding of mRNA-binding stabilizers like YB-1. (**B**) Cell stress leads to the release of nonpolysomal mRNA and induces the appearance of misfolded proteins. (**C**) The sudden excess of nonpolysomal mRNA allows aggregation-prone mRNA-binding proteins and (or) misfolded proteins to gain access to mRNA. The nonpolysomal mRNA molecules are too numerous to be protected by mRNA-binding stabilizers. (**D**) Aggregation of nonpolysomal mRNA takes place in the cytoplasm owing to homo-and heteromultimeric aggregation among aggregation-prone mRNA-binding proteins and misfolded proteins which are bound to mRNA.

The cytoplasm of mammalian cells is a crowded RNA/protein and organelle mixture. The protein concentration ranges from 100 to 200 mg/ml whereas that of RNA is in the 10–20 mg/ml range, depending on the metabolic/proliferative rates. Keeping soluble such a RNA/protein mixture represents a challenging task for cells notably because of macromolecular crowding, which promotes self-attraction between biomolecules via excluded-volume interactions (51). On the other hand, polysomes remain stable in the cytoplasm, and this is most probably due to the surfaces of the small and large ribosomal subunits which are largely negatively charged (52) and their roughness which reduces excluded-volume interactions. The stability of polysomes in the cytoplasm is also reinforced by the clustering of ribosomes along the mRNA strand leaving little room for aggregation. Under normal condition, the population of nonpolysomal mRNA is low compared to polysomal mRNA (53) and nonpolysomal mRNA is associated with specific proteins to form non aggregating mRNPs. For example, YB-1, a protein typically found in the nonpolysomal fraction of cell extracts (39), forms isolated free mRNP particles in the presence of mRNA (28). During cellular stress, a massive disruption of polysomes is observed with a sudden accumulation of nonpolysomal mRNA in the cytoplasm. mRNA freed from protecting ribosomes can seed the formation of mRNA-rich granules because of the tendency for mRNA-binding proteins to form homo- or heteromultimeric aggregates (9,54). Importantly, when nonpolysomal mRNA is in excess, mRNA-stabilizing proteins like YB-1 that prevent nonpolysomal mRNA aggregation under normal conditions can be outnumbered. Aggregation-prone proteins such as TIA-1 or the misfolded proteins can then gain access to mRNA and induce mRNA aggregation. An excess of nonpolysomal mRNA can then be the cause of the aggregation of self-attracting RNA-binding proteins leading to stress granule formation, as summarized in Figure 9.

Different facts from the present work support this hypothesis. (i) nanoSIMS investigations indicate that stress granules are indeed significantly enriched in RNA compared to protein (higher ^15^N:^14^N ratio, Figure 1D and E). (ii) HSP70 inhibition only induces stress granules when polysomes are dissociated by puromycin treatment (Figure 2A). (iii) When ribosomes are docked on mRNA by cycloheximide to dissociate stress granules (21), we observed the simultaneous dissociation of polyubuquitin-rich protein aggregates that were co-localized with stress granules during HSP70 inhibition (Figure 2B). (iv) Delivery of extra single-stranded polynucleotides from lipoplexes, either mRNA or ssDNA, also triggers stress granule assembly (Figure 3A and B). (v) When overexpressed, YB-1 prevents the formation of stress granules, most probably by preventing the binding of aggregation-prone proteins to mRNA (Figure 4B). (vi) As a proof of concept, YB-1 readily dissociates mRNA:TIA-1 aggregates into isolated mRNPs *in vitro* (Figure 5).

Such inhibition of stress granule assembly is probably not restricted to YB-1 and other mRNA-binding proteins can probably possess the same propensity provided that they do not trigger stress granule assembly by themselves. In line with this, it was found that overexpressing G3BP truncated from its putative self-attraction domain inhibits rather than triggers stress granule assembly (14). The results also suggest that the release of nonpolysomal mRNA from polysomes leads to the formation of stress granules when its concentration exceeds a ‘critical concentration’ which depends on the nature and severity of the stress to which cells are exposed.

As many RNA-binding proteins display both a nuclear and cytoplasmic location, their shuttling from the nucleus to the cytoplasm can be an interesting mean to dissociate stress granules. A different opinion has generally emerged due to the critical role of some nuclear RNA-binding proteins like TIA-1 or CIRP in stress granule assembly. Here we clearly show, using enucleated cells, that nuclear proteins shuttling from the nucleus to the cytoplasm is not required to form cytoplasmic stress granules (Figure 6), the proteins of the cytoplast being sufficient for this process. The shuttling of nuclear RNA-binding proteins to the cytoplasm rather correlates with stress granule dissociation (Figure 8). It may also provide a coordinated transcription/translation response, as exemplified here with the shuttling of HuR after transcription inhibition by ActD (Figure 7).

A tight regulation of the mRNA and mRNA stabilizer levels (YB-1) may be required to prevent abnormal formation of RNA granules due to prion-like RNA-binding proteins (TIA-1, CIRP). This novel view on RNA granule formation opens interesting speculations in RNA granule biology. For example, the RNA content is high in metabolic-active cells like neurons to sustain elevated protein synthesis as well as in highly proliferating cells (55). Such cells may then be more likely to form RNA granules during stress because they contain many polysomes. In agreement with this, when epithelial cells are forced to quiescence by contact inhibition, they have a low amount of mRNA and stress granule assembly is impaired in contrast with their proliferative counterparts (56). In metabolic-active neurons, disruption of the cell homeostasis may induce the dissociation of polysomes and the formation of RNA-rich aggregates containing RNA-binding proteins like TDP-43 or FUS, which may act as a critical step in neurodegenerative diseases (2,57–59).

## SUPPLEMENTARY DATA

Supplementary Data are available at NAR Online, including [1–5].

SUPPLEMENTARY DATA
