# Supplementary material for: Free mRNA in excess upon polysome dissociation is a scaffold for protein multimerization to form stress granules
Source: Nucleic Acids Res. 2014 Jul 10;42(13):8678–91. doi: 10.1093/nar/gku582 (PMC4117795; doi:10.1093/nar/gku582)
Supplement: SUPPLEMENTARY DATA [file supp_42_13_8678__index.html]

Free mRNA in excess upon polysome dissociation is a scaffold for protein multimerization to form stress granules — Free mRNA in excess upon polysome dissociation is a scaffold for protein multimerization to form stress granules — Free mRNA in excess upon polysome dissociation is a scaffold for protein multimerization to form stress granules — SUPPLEMENTARY DATA 

# Free mRNA in excess upon polysome dissociation is a scaffold for protein multimerization to form stress granules

## SUPPLEMENTARY DATA

**Files in this Data Supplement:**

- Supplementary Data
- Supplementary Video2
- Supplementary Video1
