# Supplementary material for: Free mRNA in excess upon polysome dissociation is a scaffold for protein multimerization to form stress granules
Source: Nucleic Acids Res. 2014 Jul 10;42(13):8678–91. doi: 10.1093/nar/gku582 (PMC4117795; doi:10.1093/nar/gku582)
Supplement: SUPPLEMENTARY DATA [file supp_gku582_nar-01287-2014-File011.pdf]

Legends to Supplementary figures and videos:

Figure S1: The small ribosomal subunit and mRNA are present in stress granules in contrast with the large ribosomal subunit.

A) NRK cells were exposed to 300 µM arsenite for 45 min. Cells were then labeled with antibodies raised against indicated proteins (YB-1, S6, RPL10). As previously published (1), we found that S6, a protein of the small ribosomal subunit, is more concentrated in stress granules than in the rest of the cytoplasm, even though to a lesser extent than YB-1 an mRNA-binding protein. On the other hand, RPL10, a protein of the large ribosomal subunit, is rather excluded from stress granules.
B) NRK cells were partially enucleated and exposed to arsenite (300 µM for 45 min). In situ hybridization was then performed to reveal Poly(A) mRNA and the 40S and 80S ribosomal subunits in arsenite-treated NRK cells as follows. Cell fixed with 4% PFA (in PBS-sucrose) for 30 min at 37° C. Cells were incubated with 100% ice-cold methanol for 10 minutes at -20° C, after that in ice-cold 70% ethanol for 10 minutes at -20° C, and then 1 M Tris pH 8 for 5 minutes, before addition of a Cy3-conjugated oligonucleaotides (Sigma) in the hybridization buffer (0.005% BSA, 1 mg/mL yeast RNA, 10% dextran sulphate, 25% formamide in 2XSSC) at 1 µg/µL. Ploy(T) of 40 nucleotides, 5'-AAGGATTTAAAGTGGACTCATTCCAATTAC and 5'GGATTCTGACTTAGAGGCGTTCAGTCATAA probes, were used to detect mRNA and the 18S (40S subunit) and 28S (80S subunit) rRNA in NRK cells respectively. Slides were then placed in a humidity chamber for 1 hour at 37˚C and shacked. Following hybridization, cells were washed once with 4X SSC and once with 2XSSC. NRK cells were also labeled with anti-YB-1 antibody to detect stress granules.

   The line profiles of fluorescent intensity over typical stress granules (SG) indicate a significant increase of the mRNA concentration and a moderate increase of the 40S subunit concentration. No increase of the 80S subunit concentration was detected in stress granules.

Figure S2: Quercetin induces stress granule assembly in the presence of puromycin and polyubiquitinated proteins were detected in stress granules.

A) Anti-YB1-labeled NRK cells were treated with quercetin (25 µM) in the presence or absence of puromycin (2.5 µg/mL). YB-1 is an mRNA-binding proteins which is used here as marker of mRNA.

B) NRK cells were treated with arsenite (300 µM for 45 min), 10 µM MG132 for 3 h in the presence or absence of puromycin. NRK cells were then immune-stained with anti-polyubiquitin. The results show that cytoplasmic aggregates containning polyubiquitinated protein appear after arsenite or puromycin/MG132 treatments. These aggregates are co-localized with stress granules (see figure 2).

Figure S3: Puromycin induces stress granule assembly in MG132-treated NRK cells.

A) NRK cells were continuously exposed to 10 µM MG132 for 4 h. Except for control, cells also received either cycloheximide or puromycin during the first 3h and then cycloheximide or puromycin for the next hour, as indicated. Anti-YB-1 labeling indicates that stress granules only appeared when puromycin was present during the last hour. Washing out puromycin during the last hour readily dissociates stress granules. Results are mean ± SD obtained on three different samples. $^{**}p < 0.005$ by *t* test. Puro, puromycin (2.5 µg/mL); Cyclo, cycloheximide (20 µg/mL).
B) NRK cells were continuously exposed to 10 µM MG132 for 4 h or to arsenite (300 µM for 45 min). The level of eIF2α phosphorylation at serine 51 was then probed using a specific anti-phosphorylated eIF2α antibody. We found that the phosphorylation level was significantly lower in MG132-treated than arsenite-treated cells. In a previous report, MG132 was shown to trigger stress granule assembly by itself but the initiation factor, eIF2α, was then significantly phosphorylated thus leading to polysome dissociation (2). This is apparently not the case for NRK cells under the condition presented in A), which explains why puromycin was necessary to trigger stress granule assembly after MG132 treatment.

C) NRK cells were exposed to 10 µM MG132 for indicated time and imuno-stained with anti-YB-1 (green) and anti-Vimentin (red). After 10 h exposure, the aggregation of vimentine, a typical marker of aggresomes, can be observed in the centrosomal area. The formation of aggregates in the centrosomal region made of misfolded proteins called aggresomes after MG132 treatment is a control that this chemical indeed targets the proteasome activity (3).

Figure S4: Effect of the mRNA concentration on stress granule formation due to mRNA transfection and kinetic of stress granule formation after mRNA transfection.

A) NRK cells were transfected with α-globin mRNA at varying concentration in the presence of 2.5 µg/mL puromycin using lipofectamine (Lipo) and cells were then fixed after 3 h for the immunofluorescence detection of stress granules.

B) Graph showing the appearance of stress granules with time. 1 h 30 are required to observe the first stress granules in the presence of puromycin. (1 µg/mL α-globin mRNA)

Figure S5: Effect of YB-1 silencing and GFP-YB-1 expression on stress granule assembly.

A) NRK cells were transfected with control or YB-1 siRNA duplexes for 36 h. NRK cells were then fixed (upper panel) or exposed to 300 µM arsenite for 45 min (lower panel). Anti-HuR labeling revealed the presence of stress granules in both control and YB-1 siRNA-treated cells (the cytoplasm of YB-1-silenced cells appears red in the merge, see arrows).

B) NRK cells were transfected with GFP-YB-1 or GFP plasmids for 36 h. NRK cells were then exposed to puromycin (2.5 µg/mL)/VER-155008 (10 µM) for 3 h (lower panel). Anti-YB-1 (endogenous and GFP-labeled YB-1) or anti-HuR labeling revealed that stress granule assembly was impaired in GFP-YB-1 expressing cells, in contrast with GFP-expressing cells. Let us recall that VER-155008 is a potent inhibitor of HSP70.

Figure S6: The enucleation process has no significant effect on the location of shuttling proteins (HuR, TIA-1) in NRK cells.

A) NRK cells were exposed to 1 µM cytochalasin D for 1 h and then cytochalasin D was washed away for 1 h. Anti-TIA-1 labeling show that these nucleocytoplasmic proteins did not significantly shuttle in the cell cytoplasm.
B) NRK cells were enucleated as described in Materials and Methods and labeled with anti-TIA-1 and DAPI as indicated. We then measured the mean anti-TIA-1 cytoplasmic fluorescence in both non enucleated and enucleated cells. Results are mean ± SD obtained on three different samples. No significant difference was found between enucleated and non enucleated cells
C) NRK cells were enucleated and labeled with anti-HuR. Note that the cytoplasmic level of HuR is similar in both non enucleated or enucleated cells.
D) NRK cells were partially enucleated and exposed to arsenite (300 µM for 45 min). Hybridization in situ was then performed to reveal Poly(A) mRNA, as previously described (Figure S1A). Stress granules were indeed observed in both enucleated and non enucleated cells.

Figure S7: Enucleated cells form stress granules excluded from the center of the cytoplast and stress granules are maintained after enucleation.

A) NRK cells were enucleated, exposed to 10 µM VER-155008 plus 2.5 µg/mL puromycin for 1 h 30 min and immune-stained with anti-YB-1 and anti-tubulin. Stress granules formed in enucleated cells are located at the cell periphery and tend to avoid the enucleated cell center.
B) NRK cells were treated with VER15508 plus puromycin 1 h prior to and during the enucleation procedure. VER15508 plus puromycin was also present in the incubation buffer after the cytochalasin D removal for 1 h. Coexisting enucleated and non enucleated cells were then immune-stained with DAPI (blue) and anti- YB-1(green). Stress granules can be observed in both enucleated and non enucleated cells.

Figure S8: HuR is not critical for stress granule assembly and its overexpression impairs stress granule assembly.

A) NRK cells were transfected with RFP-G3BP (left) or HuR (right) plasmid for 36 h. HuR overexpression in NRK cells, in contrast with RFP-G3BP expression, does not lead to stress granules assembly by itself. Image showing the distribution of YB-1 in the same area in HuR-overexpressing cells is provided as control.

B) NRK cells were transfected with control or HuR siRNA duplexes for 36 h and then exposed to 300 µM arsenite for 45 min. The presence of stress granules can be observed in both control and HuR siRNA-treated cells. (HuR-silenced cells appear with a dark nucleus in the merge see arrow)

C) After arsenite stress, higher cytoplasmic level of HuR correlates with an inhibition of stress granule assembly, although the effect was less clear than that obtained with YB-1 (figure 4B). These results are however difficult to interpret since HuR positively regulates the expression of TIA-1 (4) and probably changes the expression of other RNA-binding proteins.

D) NRK cells were treated with ActD (5 µg/mL for 2 h) and labeled with anti-TIA-1. The translocation of TIA-1 from the nucleus to the cytoplasm can be observed following the ActD treatment.

Figure S9: Short ActD treatment does not significantly impact the global mRNA level and the phosphorylation of eIF2α upon arsenite treatment.

A) The level of mRNA was estimated via in situ hybridization using cy3-labeled Poly(T) oligonucleotides as described in figure S1. 5µg /mL ActD for 2 h does not significantly decrease the level of mRNA while it is sufficient to impair stress granule assembly. In line with this, the half-life of most mRNAs is comprised between 5 to 15 h (5). In addition, stress granule assembly is not inhibited in enucleated cells treated with ActD (figure 7C), which indicates that changes in the mRNA level probably not explain the observed behavior in ActD treated-cells.

B) NRK cells were exposed to indicated treatments (arsenite 45 min, 300 µM; ActD 5µg/ml). Both western blotting and immunofluorescence with antibodies directed against indicated proteins show that eIF2α was significantly phosphorylated after arsenite exposure in ActD-pretreated cells.

Figure S10: Screening of the effect of various kinase and phosphate inhibitors on HuR translocation. NRK cells were treated for 1 h 30 with the following chemicals:

- 2 mM Vanadate, tyrosine phosphate inhibitor
- 10 µM U1026, MEK inhibitor
- 50 mM NaF, serine/theorine phosphate inhibitor
- 10 µM SB203580, P38 MAPK inhibitor
- 5 µM PKRi, PKR kinase inhibitor (C16)

- 10 µM PD0325901, MEK inhibitor
- 20 µM SP600125, JNK inhibitor
- 0.3 µM Okadaic Acid, serine/theorine phosphate inhibitor
- 10 µM CDKi, CDK1/5 inhibitor
- 40 µM Hemin, HRI kinase inhibitor
- 20 µM H89, PKA kinase inhibitor

  Cells were fixed and labeled with anti-YB-1 (green), HuR (red)

Figure S11: PKRi induces the translocation of nuclear TIA-1 and arsenite-preconditioning inhibits stress granules assembly.

A) NRK cells were exposed to arsenite (300 µM, 45 min) with or without PKRi (10 µM) 1 h prior to and during arsenite treatment.
B) NRK cells were pretreated with arsenite (200 µM for 1 h) and then allowed to recover for 3 h. Non pretreated or pretreated cells were then exposed to arsenite and labeled with anti-YB-1 for immuno-fluorescence imaging.

Video 1: Time-Lapse videomicroscopy of stress granule assembly in puromycin-treated NRK cells after HSP70 inhibition

NRK cells were transfected with a GFP-YB-1 encoding plasmid (0.5 µg/mL) and exposed to 10 µM VER-155008 plus 2.5 µg/mL puromycin. Fluoresœnce images were collected at 1 min intervals for 70 min beginning 10 min after the addition of VER-155008 plus puromycin. We selected only cells displaying low levels of GFP-YB-1 expression since higher expression levels inhibit stress granule assembly (see figure 4).

Video 2: Time-Lapse Videomicroscopy of stress granule disassembly after cycloheximide exposure.

NRK cells were transfected with a GFP-YB-1 encoding plasmid (0.5 µg/mL) and exposed to 10 µM VER-155008 plus 2.5 µg/mL puromycin for 2 h. Cycloheximide (20 µg/mL) was then added to the incubation buffer. Fluorescence images were collected at 3 min intervals for 63 min.

References of supplementary files:

1. Kedersha N, *et al.* (2002) Evidence that ternary complex (eIF2-GTP-tRNA(i)(Met))-deficient preinitiation complexes are core constituents of mammalian stress granules. (Translated from eng) *Mol Biol Cell* 13(1):195-210 (in eng).
2. Mazroui R, Di Marco S, Kaufman RJ, & Gallouzi IE (2007) Inhibition of the ubiquitin-proteasome system induces stress granule formation. (Translated from eng) *Mol Biol Cell* 18(7):2603-2618 (in eng).
3. Johnston JA, Ward CL, & Kopito RR (1998) Aggresomes: a cellular response to misfolded proteins. (Translated from eng) *J Cell Biol* 143(7):1883-1898 (in eng).
4. Kawai T, *et al.* (2006) Translational control of cytochrome c by RNA-binding proteins TIA-1 and HuR. (Translated from eng) *Mol Cell Biol* 26(8):3295-3307 (in eng).
5. Schwanhausser B, *et al.* (2011) Global quantification of mammalian gene expression control. (Translated from eng) *Nature* 473(7347):337-342 (in eng).

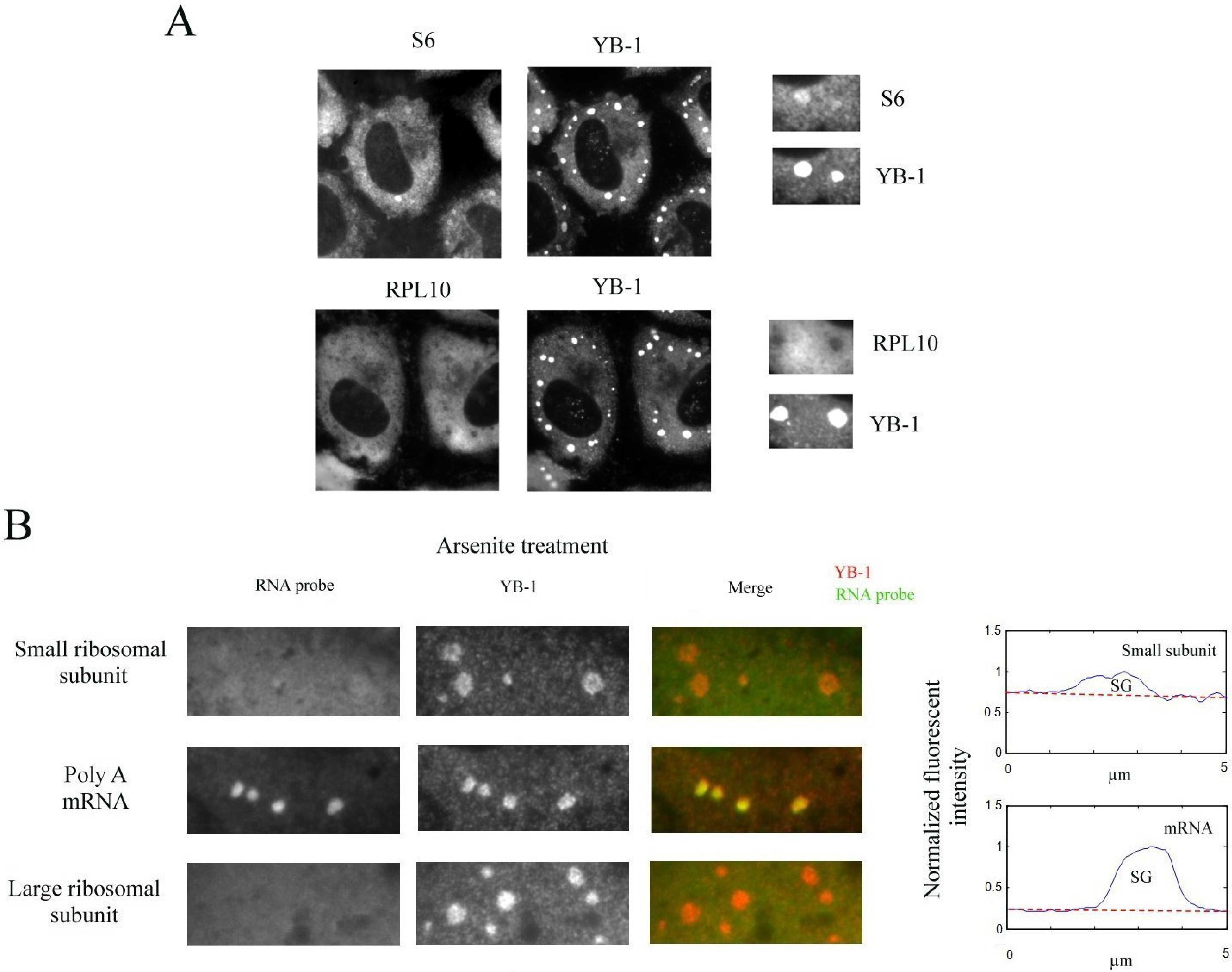
A
S6
YB-1
S6
YB-1
RPL10
YB-1
RPL10
YB-1
B
Arsenite treatment
RNA probe
YB-1
Merge
YB-1
RNA probe
Small ribosomal subunit
Poly A mRNA
Large ribosomal subunit
Normalized fluorescent intensity
Small subunit
SG
mRNA
SG
μm
μm
0
5
0
0.5
1
1.5

Figure S1

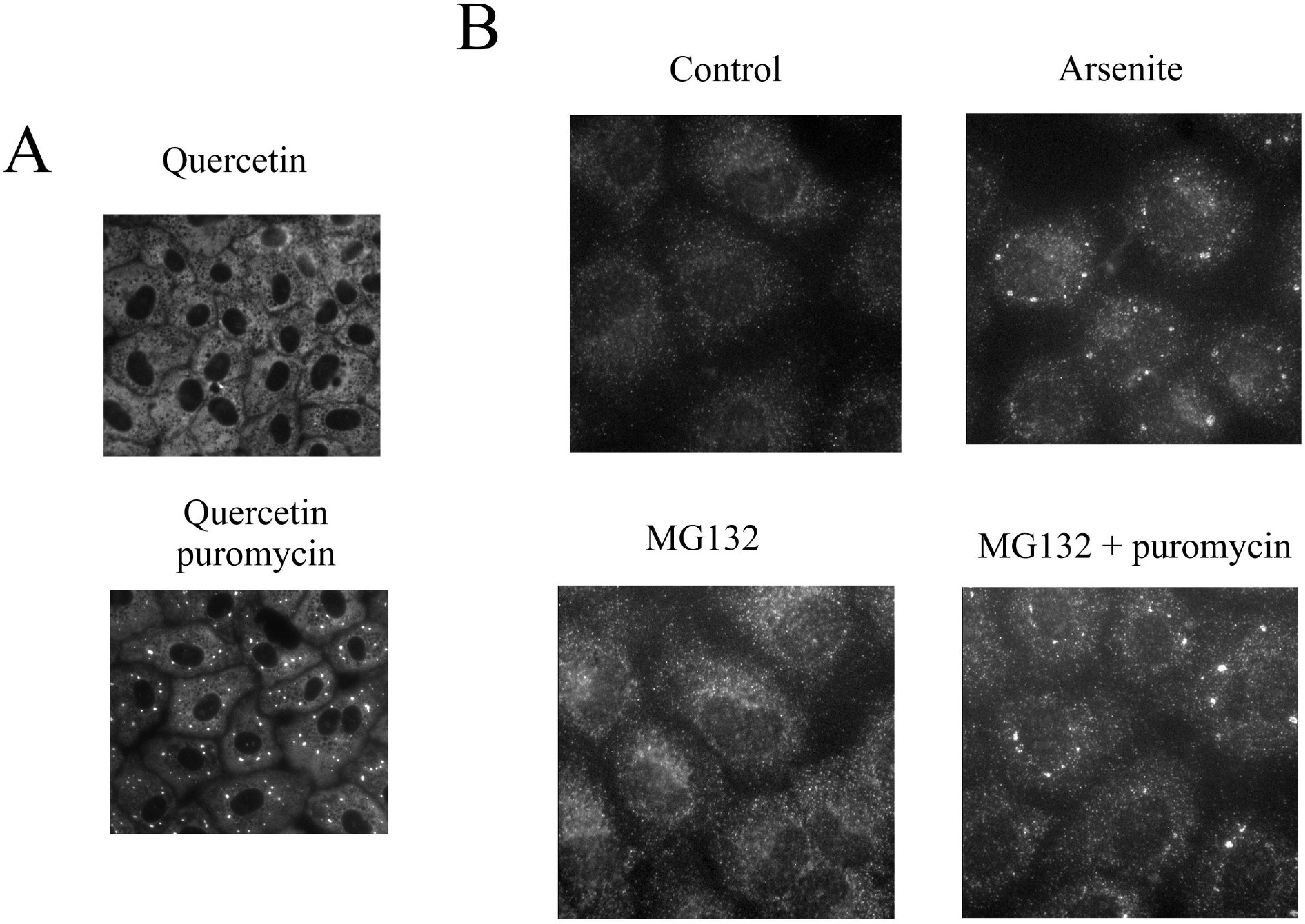
A
Quercetin
Quercetin
puromycin
B
Control
Arsenite
MG132
MG132 + puromycin

Figure S2

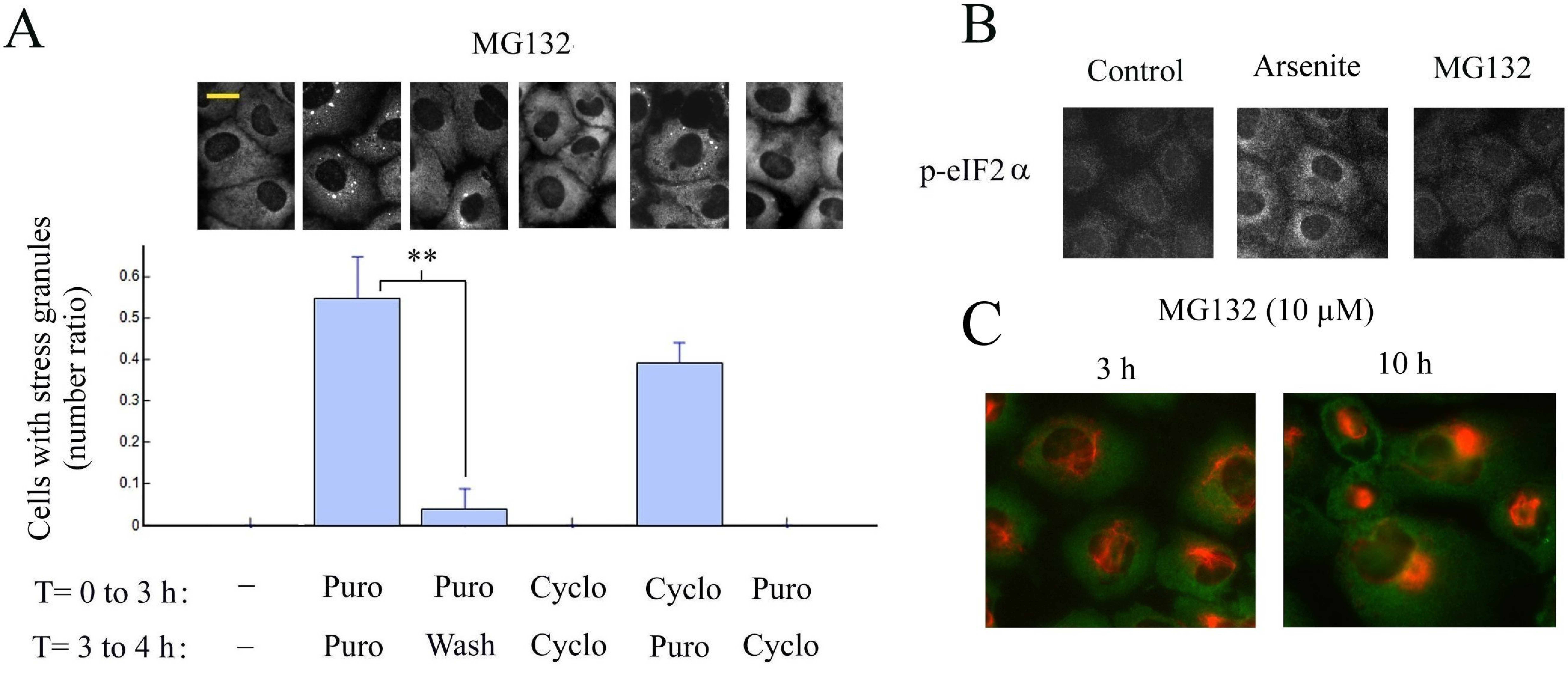

A
MG132
Cells with stress granules (number ratio)
0.6
0.5
0.4
0.3
0.2
0.1
0
**
T= 0 to 3 h:
–
Puro
Puro
Cyclo
Cyclo
Puro
T= 3 to 4 h:
–
Puro
Wash
Cyclo
Puro
Cyclo
B
Control
Arsenite
MG132
p-eIF2 α
C
MG132 (10 μM)
3 h
10 h

Figure S3

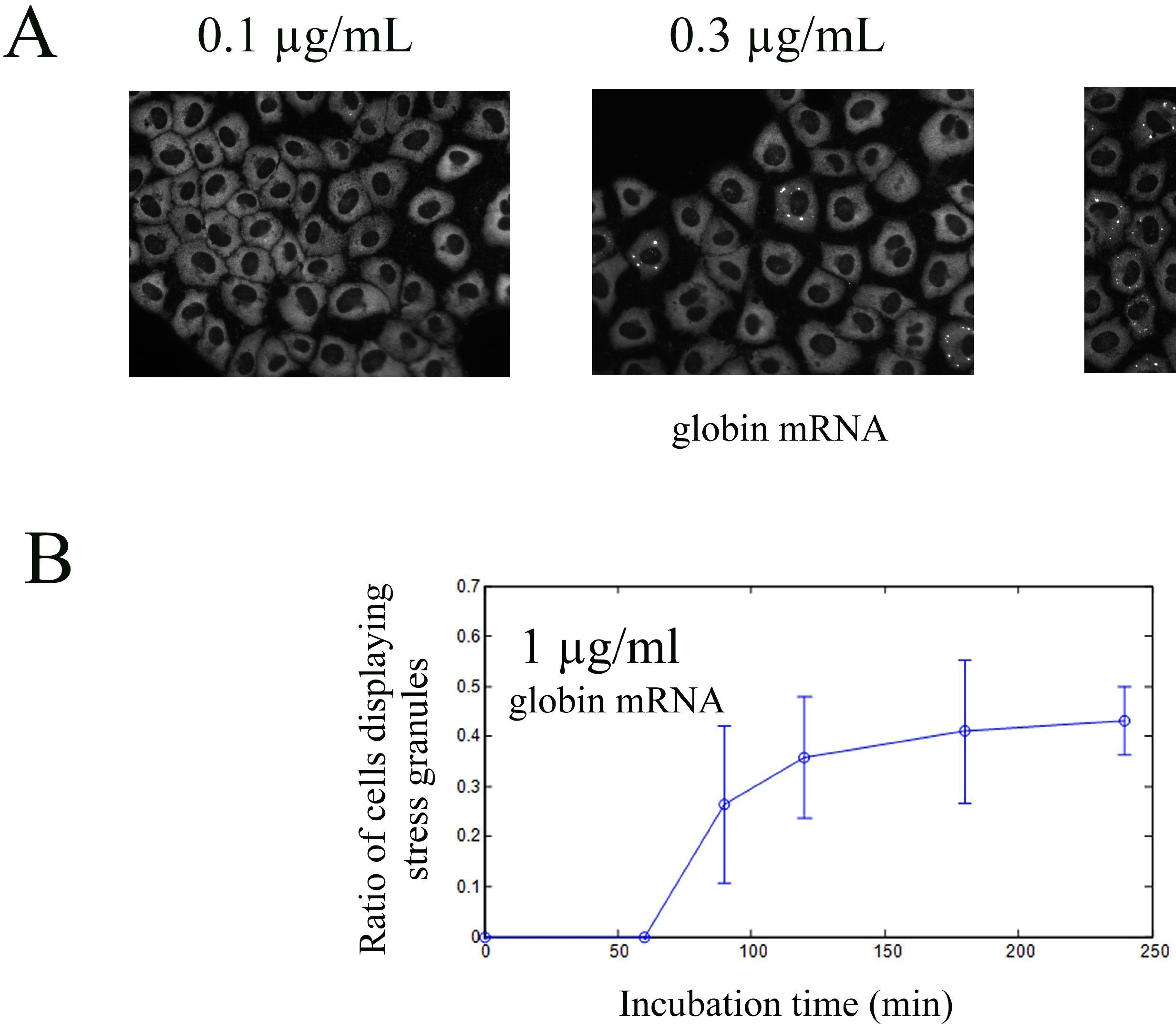
A
0.1 µg/mL
0.3 µg/mL
1 µg/mL
globin mRNA
B
1 µg/ml
globin mRNA
Ratio of cells displaying stress granules
Incubation time (min)
0
0.1
0.2
0.3
0.4
0.5
0.6
0.7
0
50
100
150
200
250

Figure S4

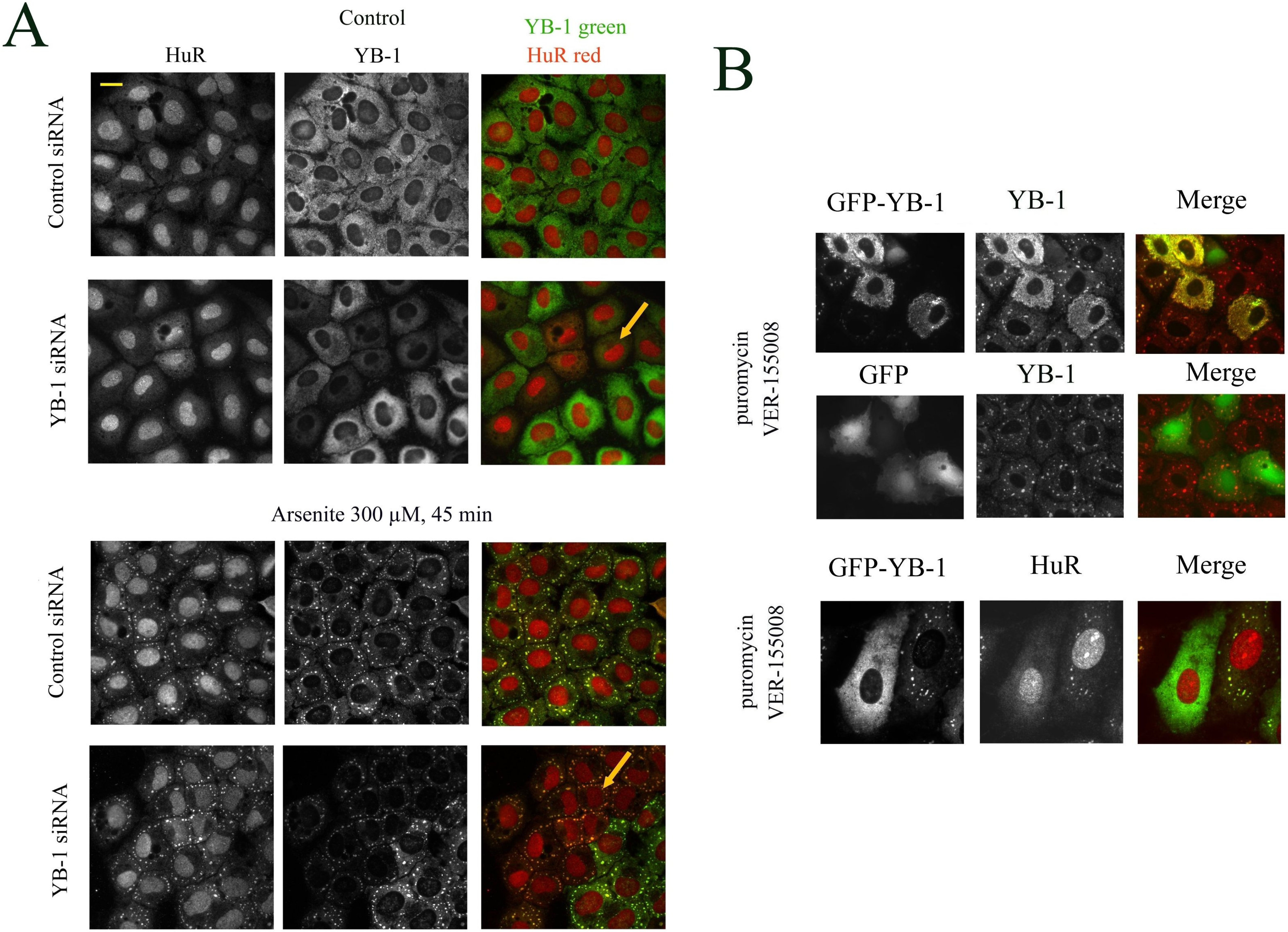
A
Control
HuR
YB-1
YB-1 green
HuR red
Control siRNA
YB-1 siRNA
Arsenite 300 µM, 45 min
Control siRNA
YB-1 siRNA
B
GFP-YB-1
YB-1
Merge
GFP
YB-1
Merge
puromycin
VER-155008
GFP-YB-1
HuR
Merge
puromycin
VER-155008

Figure S5

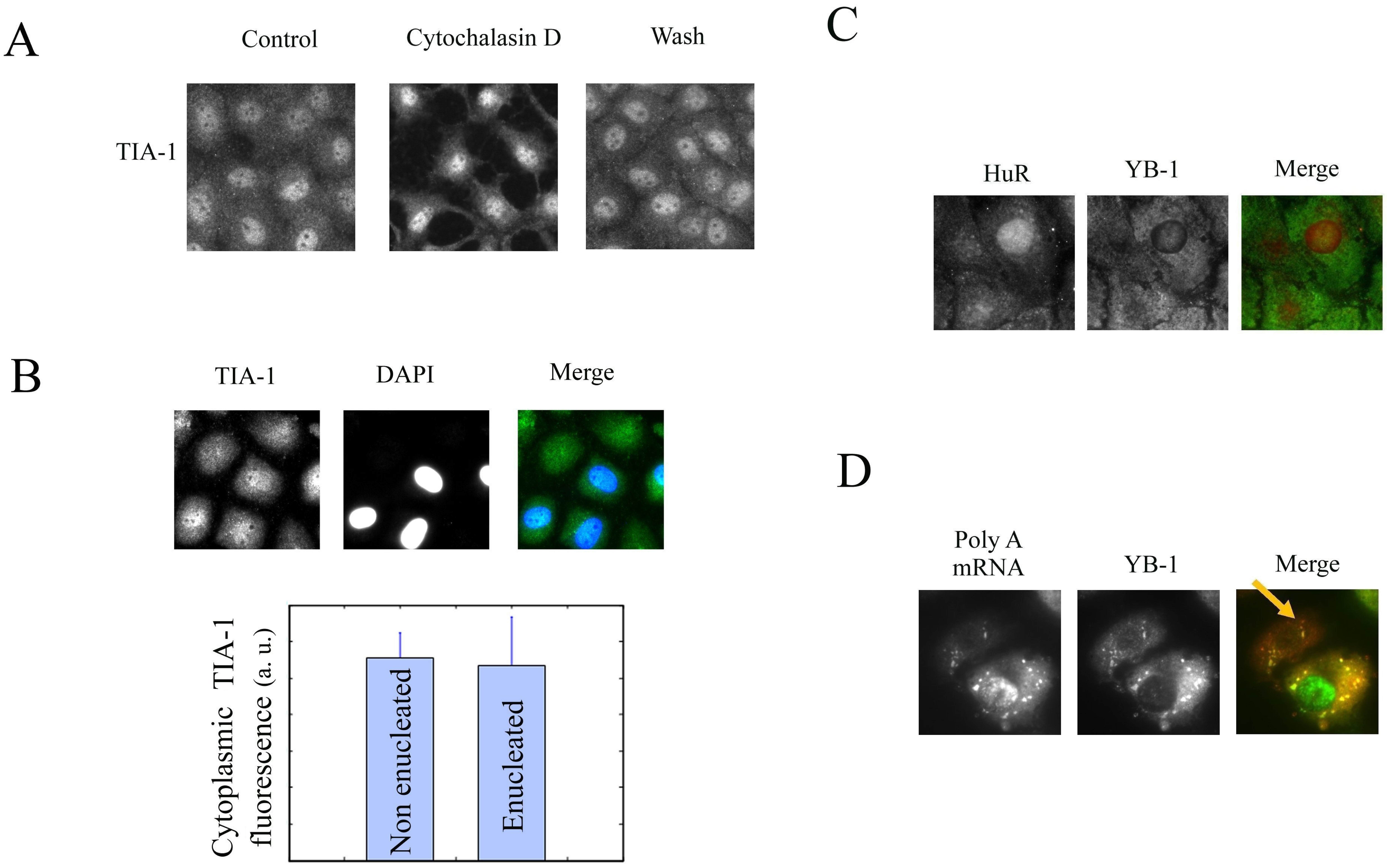

A
Control
Cytochalasin D
Wash
TIA-1
B
TIA-1
DAPI
Merge
Cytoplasmic TIA-1 fluorescence (a. u.)
Non enucleated
Enucleated
C
HuR
YB-1
Merge
D
Poly A mRNA
YB-1
Merge

Figure S6

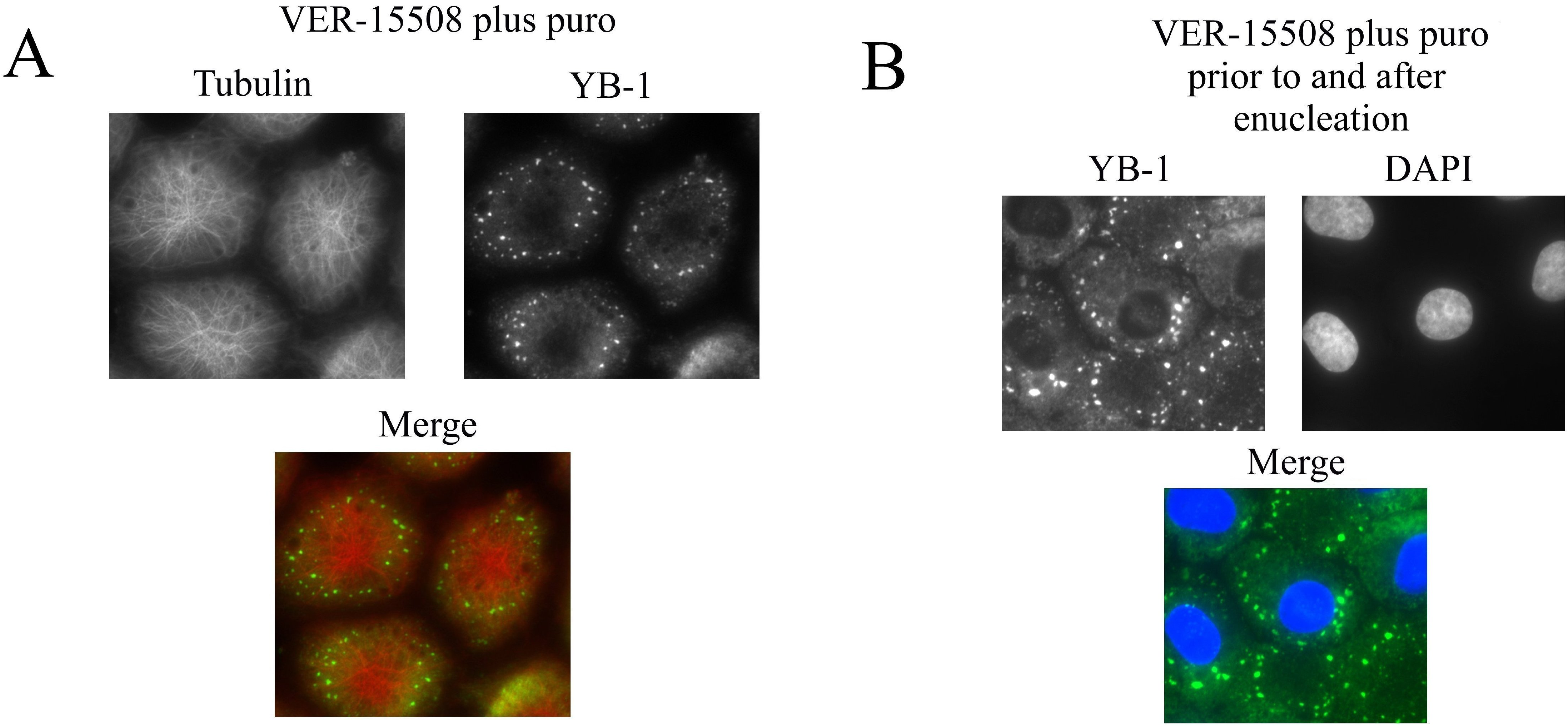
A
VER-15508 plus puro
Tubulin
YB-1
Merge
B
VER-15508 plus puro prior to and after enucleation
YB-1
DAPI
Merge

Figure S7

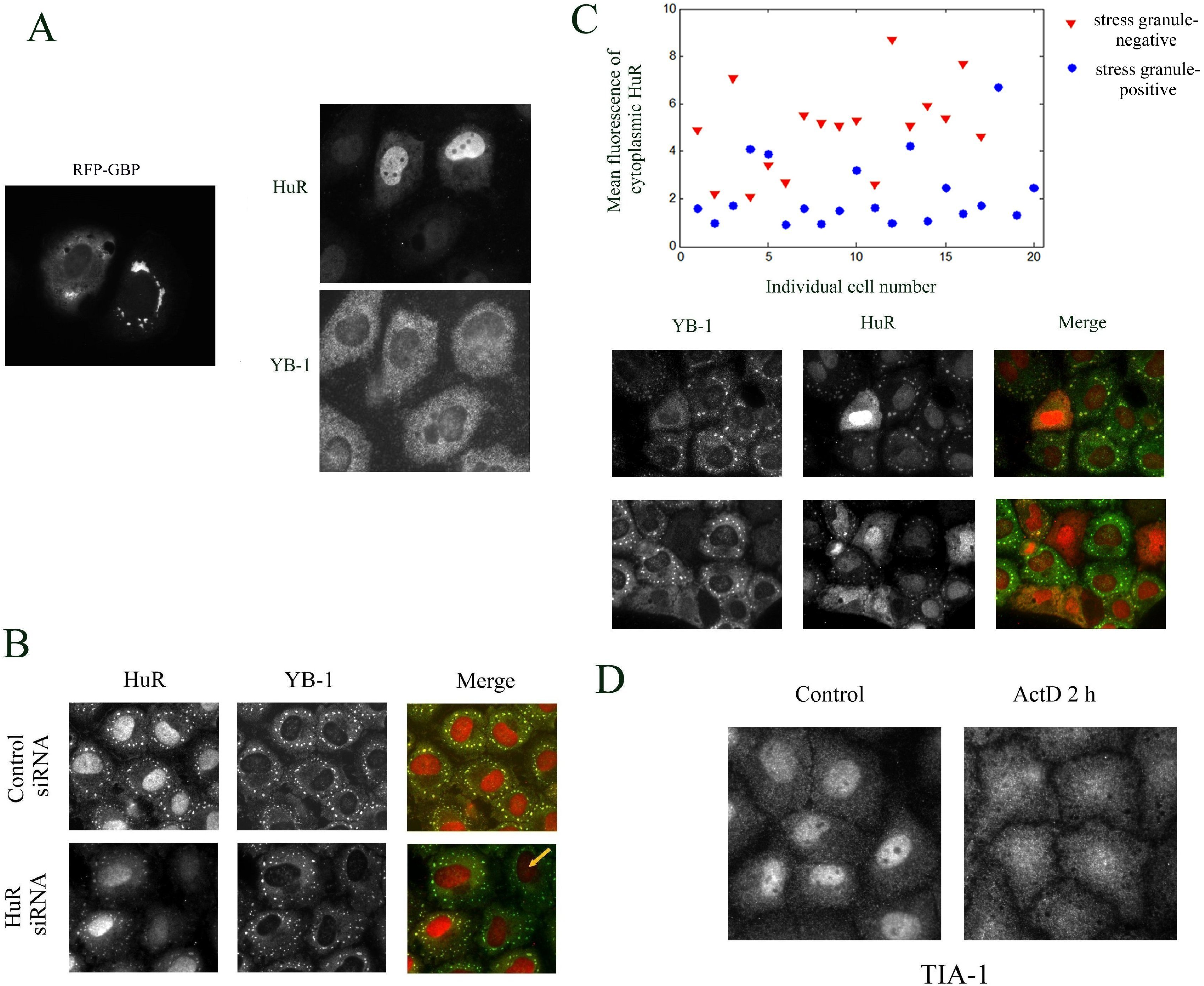
A
RFP-GBP
HuR
YB-1
C
Mean fluorescence of cytoplasmic HuR
Individual cell number
stress granule-negative
stress granule-positive
YB-1
HuR
Merge
B
HuR
YB-1
Merge
Control siRNA
HuR siRNA
D
Control
ActD 2 h
TIA-1

Figure S8

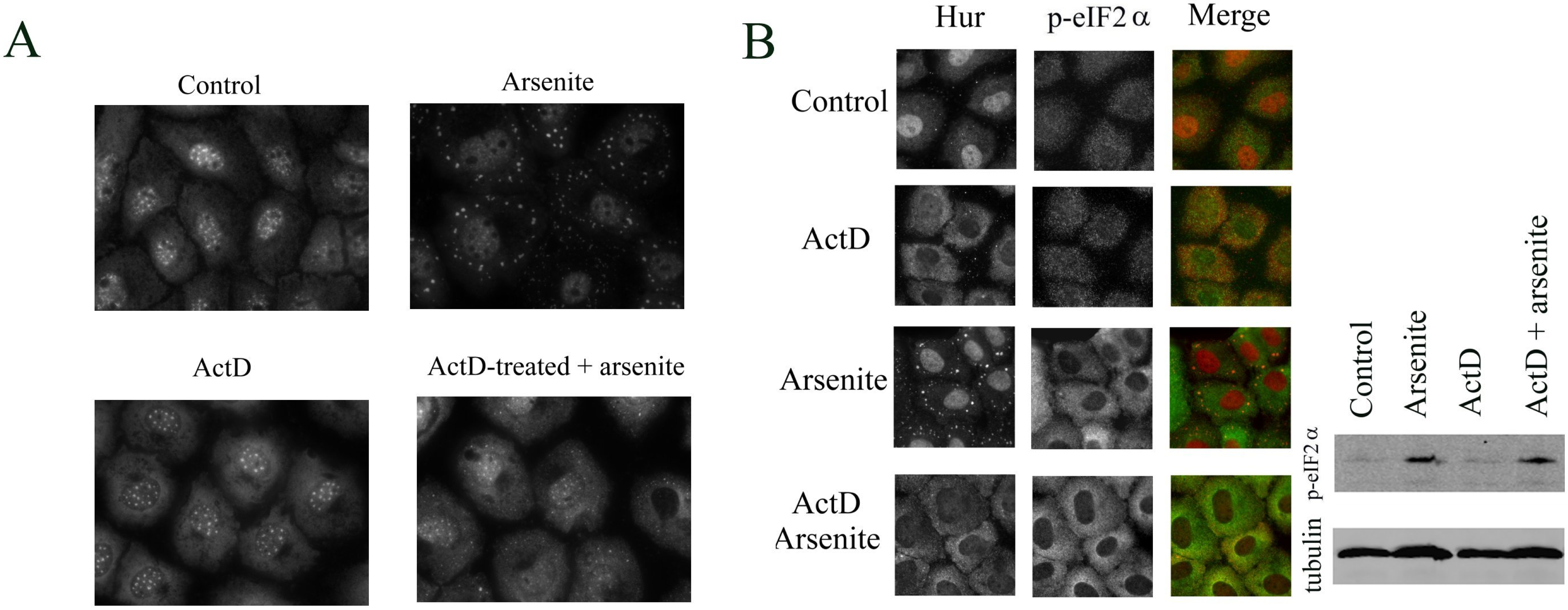
A
Control
Arsenite
ActD
ActD-treated + arsenite
B
Hur
p-eIF2 α
Merge
Control
ActD
Arsenite
ActD
Arsenite
Control
Arsenite
ActD
ActD + arsenite
p-eIF2 α
tubulin

Figure S9

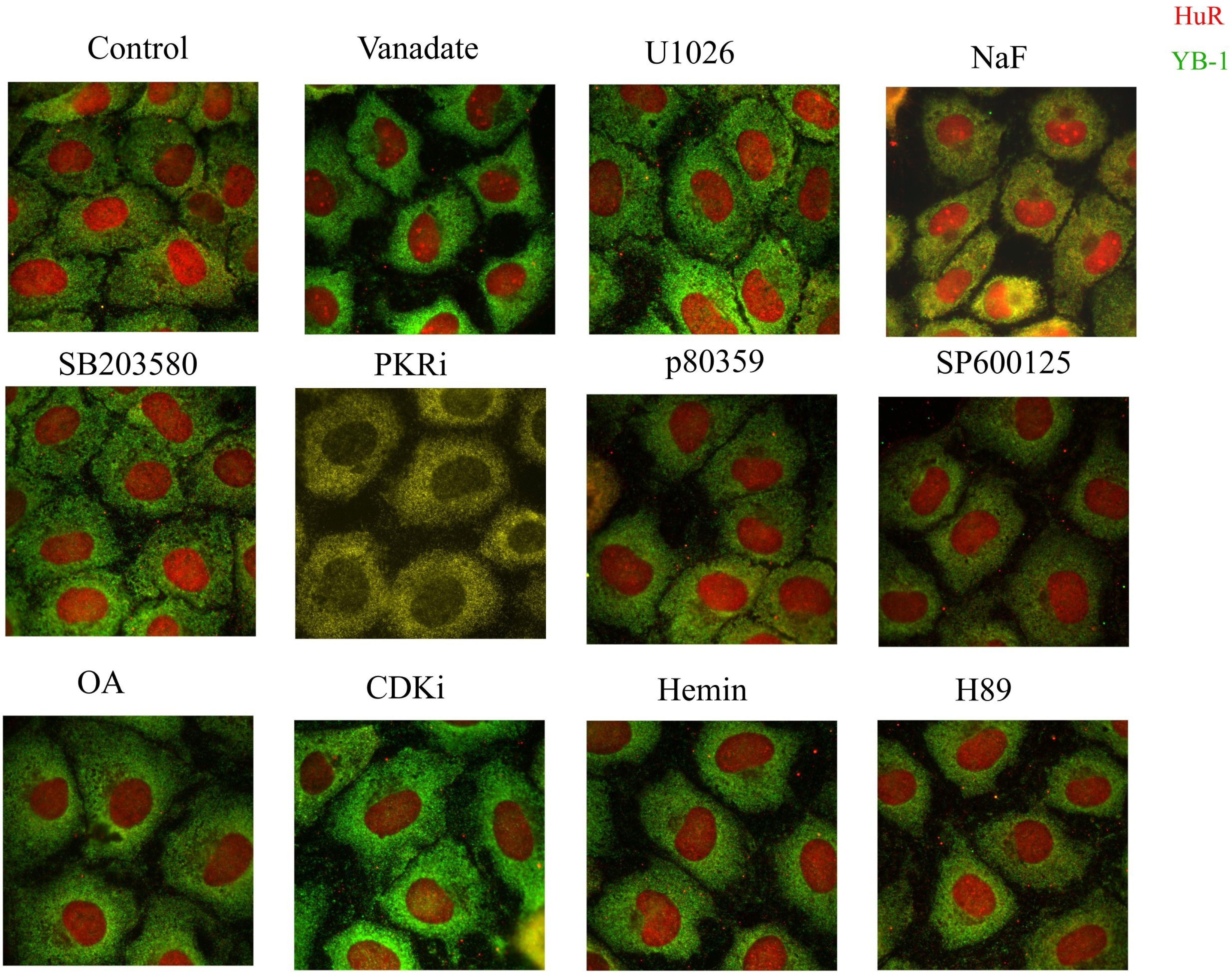
HuR
YB-1
Control
Vanadate
U1026
NaF
SB203580
PKRi
p80359
SP600125
OA
CDKi
Hemin
H89

Figure S10

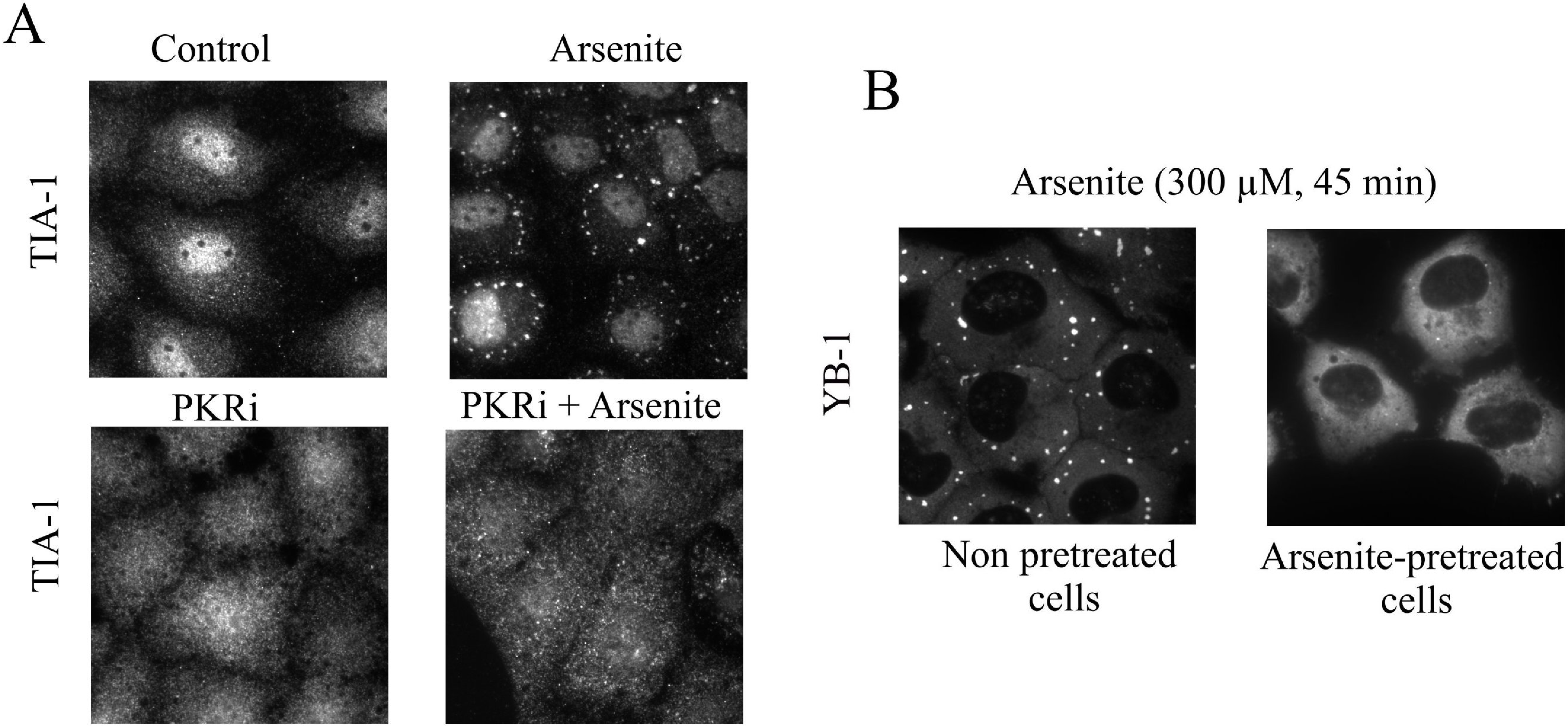
A
Control
Arsenite
TIA-1
PKRi
PKRi + Arsenite
TIA-1
B
Arsenite (300 μM, 45 min)
YB-1
Non pretreated cells
Arsenite-pretreated cells

Figure S11
